# Improvement in Human Immune Function with Changes in Intestinal Microbiota by *Salacia reticulata* Extract Ingestion: A Randomized Placebo-Controlled Trial

**DOI:** 10.1371/journal.pone.0142909

**Published:** 2015-12-02

**Authors:** Yuriko Oda, Fumitaka Ueda, Masanori Utsuyama, Asuka Kamei, Chihaya Kakinuma, Keiko Abe, Katsuiku Hirokawa

**Affiliations:** 1 Pharmaceutical and Healthcare Research Laboratories, Research and Development Management Headquarters, FUJIFILM Corporation, Kanagawa, Japan; 2 Institute for Health and Life Science, Tokyo, Japan; 3 Department of Comprehensive Pathology, Tokyo Medical & Dental University, Tokyo, Japan; 4 Kanagawa Academy of Science and Technology, Kanagawa, Japan; 5 Department of Applied Biological Chemistry, Graduate School of Agricultural and Life Science, The University of Tokyo, Tokyo, Japan; Rush University, UNITED STATES

## Abstract

Plants belonging to the genus *Salacia* in the *Hippocrateaceae* family are known to inhibit sugar absorption. In a previous study, administration of *Salacia reticulata* extract in rats altered the intestinal microbiota and increased expression of immune-relevant genes in small intestinal epithelial cells. This study aimed to investigate the effect of *S*. *reticulata* extract in human subjects by examining the gene expression profiles of blood cells, immunological indices, and intestinal microbiota. The results revealed an improvement in T-cell proliferation activity and some other immunological indices. In addition, the intestinal microbiota changed, with an increase in *Bifidobacterium* and a decrease in *Clostridium* bacteria. The expression levels of many immune-relevant genes were altered in blood cells. We concluded that *S*. *reticulata* extract ingestion in humans improved immune functions and changed the intestinal microbiota.

***Trial Registration***: UMIN Clinical Trials Registry UMIN000011732

## Introduction

Root and stem parts of the plants of the genus *Salacia* in the *Hippocrateaceae* family, including *Salacia reticulata* and *S*. *oblonga*, have been used for generations as Ayurvedic medicine that are effective for treating diabetes [1, 2]. These plants contain characteristic chemical components, such as salacinol, kotalanol, neosalacinol, neokotalanol, and mangiferin [3, 4]. Salacinol, kotalanol, neosalacinol, and neokotalanol are known to suppress blood glucose levels in glucose-loaded rats [5, 6]. These compounds have been shown to have α-glucosidase inhibitory activity *in vitro* [3, 4, 7, 8].

*Salacia reticulata* extract (SRE) contains polyphenols, such as catechin and water-soluble dietary fibers [9]. These edible plants have been confirmed to provide health maintenance effects in humans, such as suppressing blood glucose level elevation, decreasing body weight, and reducing LDL cholesterol levels [10, 11]. With their safety having been established, these plants have attracted increasing attention in recent years as safe and useful foods [12–15].

The immune system has an important role in maintaining a healthy condition; however, its activity begins to decline after puberty, although individual variation s in the start and magnitude of age-related decline in immune function are prominent. According to the Vital Statistics 2013 by the Ministry of Health, Labour and Welfare, the 4 major causes of death in Japan are cancer, heart diseases, cerebrovascular diseases, and infection. The occurrence of these diseases is closely associated with immunological decline, particularly in the elderly population. Thus, many individuals and groups are searched regarding for methods or materials for immunological restoration.

Recent reports have indicated that the entire immune system is closely related to the intestinal condition. In particular, overall and intestinal microbiotas are also affected by age [16–18]. Thus, many experiments have been performed to evaluate the effects of dietary fibers, prebiotic/probiotics, yogurt fermented with lactobacilli, and glucan and others on the immune system and microbiota, although the results were not always convincing [19–22].

Previously, we performed gene expression analysis of the intestinal epithelium and measured the intestinal microbiota in rats fed the SRE powder [23]. We confirmed that the expression levels of genes associated with immunologically relevant cells, particularly those associated with cell-mediated immunity, were increased in the intestinal epithelium after SRE administration. Also, we demonstrated that SRE administration altered the intestinal microbiota in rats. However, rats differ from humans in their intestinal microbiota composition and lifestyle habits; therefore, the effects of *S*. *reticulata* on immune function and intestinal microbiota in humans remain unknown.

In this study, we aimed to reveal the effects of SRE in healthy adults with mildly reduced immunity. We conducted an ingestion test of SRE using a randomized, double-blind, parallel-group, placebo-controlled design to evaluate the immunological effect of SRE in human blood and feces. Changes in immune strength and intestinal microbiota were evaluated by measuring various immunological indices in the blood and by intestinal microbiota profiling. In subjects who consumed SRE, gene expression analysis of blood was performed by DNA microarrays in an attempt to evaluate the effect of *S*. *reticulata* on the expression of various genes.

The ingested SRE induced changes in the intestinal microbiota, improved T-cell proliferation index and other various immunological indices known to decline with aging, and elevated the expression levels of immune-relevant genes. These results demonstrated that in humans, SRE altered the intestinal microbiota and acted on the immune system to enhance immune function.

## Methods

### Preparation of the SRE powder

The stems and roots of *S*. *reticulata* harvested in Sri Lanka by Eco Tech Create 21 Co., Ltd. (Sri Lanka) were purchased from Eco Tech Create 21 Co., Ltd. (Japan). The stems and roots were subjected to botanical comparative identification with reference samples in the herbarium by thin-layer chromatography fingerprinting at the Industrial Technology Institute in Sri Lanka and were confirmed to belong to *S*. *reticulata*. The stem and root parts were dried and processed into chips. After sufficient drying, the chips were extracted into hot water (90°C) for 1 h [chip:water = 1:9 (w/w)]. After removal of the chips by filtration, the liquid extract was cooled and subjected to spray drying in an ADL-310 spray dryer (Yamato Science Co., Ltd., Tokyo, Japan) overnight to obtain a powdery extract, which was stored at 4°C. The SRE powder contained 61% carbohydrates, 17% polyphenols, 12% ash content, 5% water, 4% proteins, and 0.6% lipids. Salacinol and kotalanol concentrations in the SRE powder were 4.17 ± 0.13 and 2.26 ± 0.12 μg/mg, respectively.

### Test food

The test food was prepared in a tablet form (Table 1). The SRE powder was ingested by the subjects as 60 mg SRE (one tablet) before both breakfast and lunch and 120 mg SRE (two tablets) before dinner with water or plain hot water to match the volume of each meal, according to the 2012 National Health and Nutrition Survey in Japan published by the Japanese Ministry of Health, Labor, and Welfare. A placebo was formulated using materials with no effects on the intestinal environment or microbiota. The placebo tablets were indistinguishable from the SRE tablets in appearance and were ingested by the subjects in the same manner as the SRE tablets. Test food consumption was monitored via a Web-based diary, and subjects whose consumption rate was <90% were excluded.

**Table 1:** Formulae of test and placebo tablet formulations.

	*Salacia reticulata* (mg)	Placebo (mg)
***S*. *reticulata* extract**	60.00	—
**Calcium carbonate**	6.00	—
**Crystalline cellulose**	172.75	175.00
**Sucrose fatty acid ester**	7.50	—
**Silica dioxide**	3.75	2.50
**Erythritol**	—	50.00
**Calcium stearate**	—	2.50
**Color adjuster**	—	20.00
**Total**	250.00	250.00

### Study design

The study used a randomized, double-blind, parallel-group, placebo-controlled design and was conducted between October 1, 2013 and October 28, 2013 (4 weeks) in Tokyo, Japan. Data for body height, body weight, body fat, blood pressure, pulse, biochemistry tests, immunological index, and intestinal microbiota before and after ingestion of the test food were obtained for all subjects. Whole blood was collected for gene expression analysis (S1 CONSORT Checklist and S1 Protocol).

### Subjects

The subjects included healthy males aged 50–60 years. To match the lifestyle, only sedentary workers were included. In total, 43 volunteers who provided written informed consent to participate in the present study were screened, and those with a high immune strength score (IV or above) or hemoglobin A1c (HbA1c) levels outside the normal range (Japan Diabetes Society value <5.8%) were excluded [24]. In total, 32 subjects with a low immune strength score (<IV) 32were included. The subjects were randomly divided into two groups such that the groups were uniform in the immune strength score, T-cell proliferation capacity, and T-cell count; the resulting groups were assigned to either of the test foods. The allocation table was maintained by the person in charge of allocation until the Key code break meeting.

This study was conducted in compliance with the Declaration of Helsinki and with the approval of the ethics review committee of the Shiba Palace Clinic. The outline of this study was included in the UMIN Clinical Trials Registry as UMIN000011732. The subjects were forbidden to consume food and drugs that affect the intestinal microbiota, such as prebiotic/probiotics (e.g.,., *Bifidobacterium*, *Lactobacillus*, oligosaccharides, fibers), yogurt, and antibiotics from 1 week before screening but otherwise instructed to maintain their usual lifestyle (dietary, life rhythm, and sleep time). Any medication used was recorded in the Web-based diary.

### Blood collection

Blood sample (2.5 ml) was collected in a PAXgene RNA blood collection tube (Becton-Dickinson, Mountain View, California, USA) and used for gene expression analysis. Another blood sample (2 ml) was collected in a blood collection tube containing ethylenediaminetetraacetic acid-2K and used for flow cytometry, blood count, differential leukocyte count, and lymphocyte subpopulation cell count. Another blood sample (8 ml) was collected in a blood collection tube for mononuclear cell separation (Becton–Dickinson) and used for the isolation of mononuclear cells for immunological analysis. Separately, blood biochemical tests were performed for screening (HbA1c) and safety checking.

### Gene expression analysis

#### Gene expression (DNA microarray assay)

In the SRE group, seven subjects (14 samples) were randomly chosen and subjected to pre- and post-ingestion gene expression analysis.

Blood samples collected in the PAXgene RNA blood collection tubes were stored at −80°C. Total RNA was extracted from the samples using the PAXgene Blood RNA Kit (QIAGEN, Hilden, Germany). Globin RNA was removed from the samples using the GLOBINclear Kit (Life Technologies, Gaithersburg, MD, USA) prior to microarray experiments. cDNA synthesis from the blood-derived total RNA, cRNA synthesis, and labeling and fragmentation of the labeled cRNA was performed according to the Affymetrix (Santa Clara, CA, USA) protocol (GeneChip^®^ 3′ IVT PLUS Reagent Kit). RNA degradation and cRNA elongation were verified by an Agilent 2100 bioanalyzer (Agilent Technologies Japan, Ltd., Tokyo, Japan). All samples had an RNA integrity number ≥7.8. The fragmented cRNA was hybridized using the GeneChip^®^ Human Genome U133 Plus 2.0 Array (Affymetrix) at 45°C for 16 h and a Hybridization Oven 640 (Affymetrix), washed, stained by a GeneChip^®^ Fluidics Station 450 (Affymetrix), and scanned by a GeneChip^®^ Scanner 3000 to measure the gene expression levels. Image data obtained with each probe were converted to intensity values (CEL files) using GeneChip^®^ Operating Software ver1.4 (Affymetrix).

#### DNA microarray data analysis

The obtained data were normalized by the distribution-free weighting (DFW) method with R version 2.7.2 (http://cran.r-project.org/bin/windows/base/old/2.7.2/) [25]. Intergroup comparison of the DFW-normalized data was performed by the rank products method of R version 2.14.2 [26]. Probe sets with an false discovery rate (FDR) of <0.05 were extracted and subjected to gene annotation enrichment analysis on the basis of Gene Ontology (GO) data according to biological functions with DAVID ver. 6.7 (http://david.abcc.ncifcrf.gov/) using the Jackknife Fisher exact test. IDs of the probe sets used as input data were provided by Affymetrix. The functional annotation chart was analyzed on the basis of biological processes in GO, GOTERM_BP_ALL. Pathway analysis was performed by IPA (http://www.ingenuity.com/) and the abovementioned probe sets with an FDR of <0.05.

### Immunological index

Flow cytometry counting of various cells and immunological analysis of mononuclear cells for subject screening and immune function evaluation were performed on the basis of methods previously reported by Hirokawa *et al*. [24, 27, 28]. Only modifications from the original method are described below.

#### Cytokine measurement

For the isolation of blood mononuclear cells, peripheral blood was centrifuged at 3,000 rpm (Tomy LC-230; TOMY SEIKO Co., Ltd., Tokyo, Japan), and the cell layer on the gel barrier was recovered. After washing with physiological saline, the isolated mononuclear cells (1 × 10^6^) were cultured in RPMI-1640 (Gibco, Carlsbad, CA, USA) containing 10% FBS, 50 ng/ml PMA, and 500 ng/ml ionomycin for 48 h, and then cytokines (IL-1β, IL-2, IL-4, IL-5, IL-6, IL-8, IL-10, IL-12p70, and IL-17A; IFN-γ, TNF-α and TNF-β) were measured using a Flowcytomix Kit (eBioscience, San Diego, CA, USA). A Navios flow cytometer (Beckman Coulter Inc., Brea, CA, USA) was used for the measurement.

#### T-cell proliferation index

A detailed explanation of the T-cell proliferation index was reported by Hirokawa *et al*. [24]. The T-cell proliferation index was calculated according to the following equation:

$$\text{T-cell proliferation index}=\text{T-cell proliferative activity}\times \left({\text{T-cell number per mm}}^{3}/1000\right)$$

#### Immunological age

The immunological age was determined from the average and standard deviation of the pre-immunological age, as reported by Hirokawa *et al*. [29]. The pre-immunological age was calculated according to the following regression equation of the T-cell proliferation index and age:

Pre-immunological age = (2.535−T-cell proliferation index)/0.017

### Intestinal microbiota analysis by the terminal restriction fragment length polymorphism method

Intestinal microbiota measurements were performed with pre-ingestion and post-ingestion fecal samples collected and stored using a Feces Sampling Kit (TechnoSuruga Laboratory Co., Ltd., Shizuoka, Japan). Intestinal microbiota analysis was performed by TechnoSuruga Laboratory Co., Ltd. by terminal restriction fragment length polymorphism (T-RFLP) analysis (Nagashima method) [30] with some modifications. The modifications from the original method are described below.

Frozen fecal samples were suspended in GTC buffer (100 mM Tris–HCl (pH 9.0), 40 mM Tris–EDTA (pH 8.0), 4 M guanidine thiocyanate, and 0.001% bromothymol blue) and then ground using zirconia beads [5 m/s, 2 min, FastPrep 24 Instrument (MP Biomedicals, CA, USA)]. Using 100 μl of the suspension, DNA was extracted with an automated nucleic acid extraction apparatus (Precision System Science, Chiba, Japan). The reagent used for the automated nucleic acid extraction was MagDEA^®^ DNA 200 (GC; Precision System Science). FAM was used for 516F labeling of PCR primers, instead of HEX used in the reference. PCR products were purified by MultiScreen PCRμ96 plates (Millipore, Billerica, MA, USA).

Fragment analysis was performed by an ABI PRISM 3130xl genetic analyzer (Applied Biosystems, CA, USA) and GeneMapper^®^ software (Applied Biosystems). The standard size markers used were MapMarker^®^ X-Rhodamine Labeled 50–1000 bp (Bioventures, TN, USA).

### Statistical analysis

The Kolmogorov–Smirnov test was used to evaluate the immunological index and intestinal microbiota analysis data for normality. If normality was not shown, comparison between values obtained before and after ingestion of the test food was performed by the Wilcoxon signed-rank test. For intergroup comparisons, the amount of change (post-ingestion value minus the pre-ingestion value) was computed, and statistical analysis was performed by the Mann–Whitney *U*-test.

When normality was shown, comparison between values obtained before and after ingestion of the test food was performed by the paired *t*-test. For intergroup comparisons, the amount of change was computed, and *F* tests for equality of variance were performed. Significant differences were analyzed by Student’s *t*-test (homoscedasticity) or Welch’s *t*-test (heteroscedasticity). Statistical analysis was performed by R version 2.7.2 (http://cran.r-project.org/bin/windows/base/old/2.7.2/).

## Results

**Fig 1:**
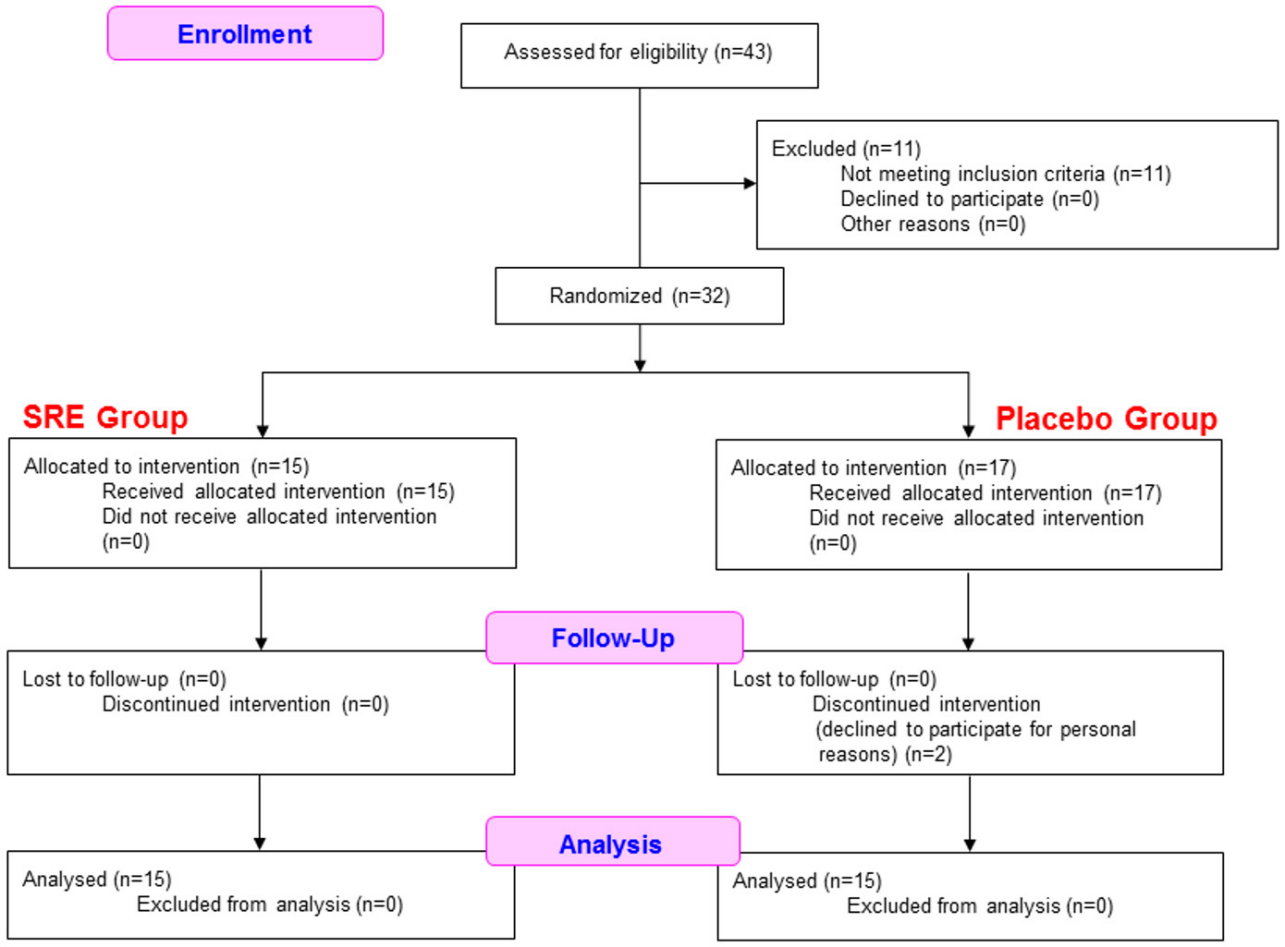
Flow and number of subjects in each phase of the study.

Fig 1 shows subject flow from screening to last assessment. Of the 32 subjects included in the present study, two declined to participate for personal reasons; the remaining 30 subjects were included in the statistical analyses. The study protocol was not modified during the study period.

Both the SRE and placebo (P) groups comprised 15 subjects, with no significant difference in age between the two groups (SRE group: 54.0 ± 0.6 years; P group: 54.1 ± 0.7 years). No measurable differences in body height, body weight, body fat, blood pressure, pulse, immunological index, and ratio of intestinal microbiota were found between the groups before the test. Neither the Web-based diary nor blood biochemistry tests suggested adverse events attributable to SRE intake. The adherence rates for the test food were >95% in both groups.

### Gene expression analysis

In gene expression analysis of the blood samples, 1017 genes were upregulated, and 1048 genes were downregulated. There were 59 GO terms with a Benjamini and Hochberg FDR-corrected *p* value of <0.01 calculated by DAVID ver. 6.7. These significantly enriched GO terms included immunity and body defense functions (S1 Table). The representative examples were immune system process (GO:0002376), immune response (GO:0006955), leukocyte activation (GO:0045321), lymphocyte activation (GO:0046649), response to biotic stimulus (GO:0009607), response to stress (GO:0006950), defense response (GO:0006952), response to stimulus (GO:0050896), and T-cell activation (GO:0042110). In addition, the extracted GO terms included those suggesting the action of intestinal bacteria, including response to bacterium (GO:0009617), response to molecule of bacterial origin (GO:0002237), and response to lipopolysaccharide (GO:0032496).

**Fig 2:**
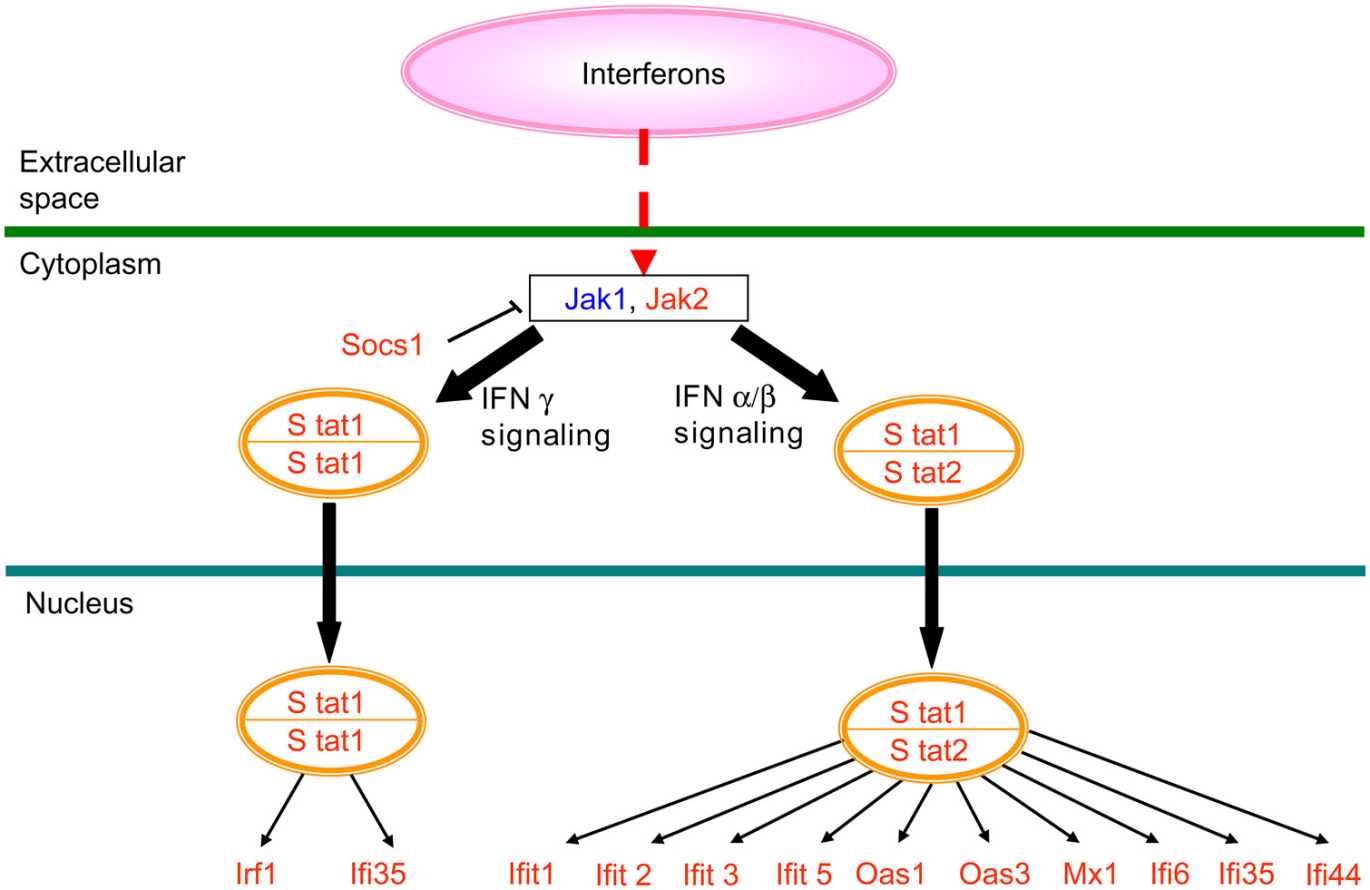
Genes whose expression was altered by *Salacia reticulata* extract (SRE) ingestion (interferon (IFN) signaling). Probe sets acquired by the rank products method are shown (FDR < 0.05). Expression levels of multiple IFN signaling-associated genes were altered, with levels of most genes being increased after SRE ingestion. Red text: upregulated genes and blue text: downregulated genes.

Based on this result, detailed analysis was performed by Ingenuity Pathways Analysis^™^. Canonical pathway analysis revealed that the interferon (IFN) signaling pathway was highly enriched in differentially expressed genes (Fig 2). The IFN signaling pathway is mediated by JAKs and STATs.

Expression of most of the genes involved in IFN signaling was enhanced (S2 Table). Detailed analysis of genes with increased expression revealed that many were involved in IFN expression, such as IFN-induced protein with tetratricopeptide repeats 1, 2, 3, and 5 (Ifit1, Ifit2, Ifit3, and Ifit5, respectively), IFN-induced protein 44 (Ifi44), and IFN-α-inducible protein 6 (Ifi6). In addition, the expression levels of genes involved in antiviral activity, such as myxovirus (influenza virus) resistance 1 (Mx1) and 2′, 5′-oligoadenylate synthetase 1 and 3 (Oas1, Oas3); toll-like receptors 1 and 5 (Tlr1, Tlr5) that recognize bacteria; and histamine *N*-methyltransferase (Hnmt), which acts in histamine decomposition, increased (S3 Table).

Although many immune-relevant genes were also found among downregulated genes, they also included prostaglandin D2 synthase (Ptgds) and prostaglandin D2 receptor (Ptgdr), which are involved in allergic reactions; granulysin (Gnly), which is expressed in activated cytotoxic T cells (CTL) and NK cells; and Janus kinase 1 (Jak1) and RAR-related orphan receptor A (Rora), which are related to T-helper 17 cells (Th17). In essence, inflammation-related genes, except for IFN-related genes, were downregulated (S4 Table).

### Immunological index

**Fig 3:**
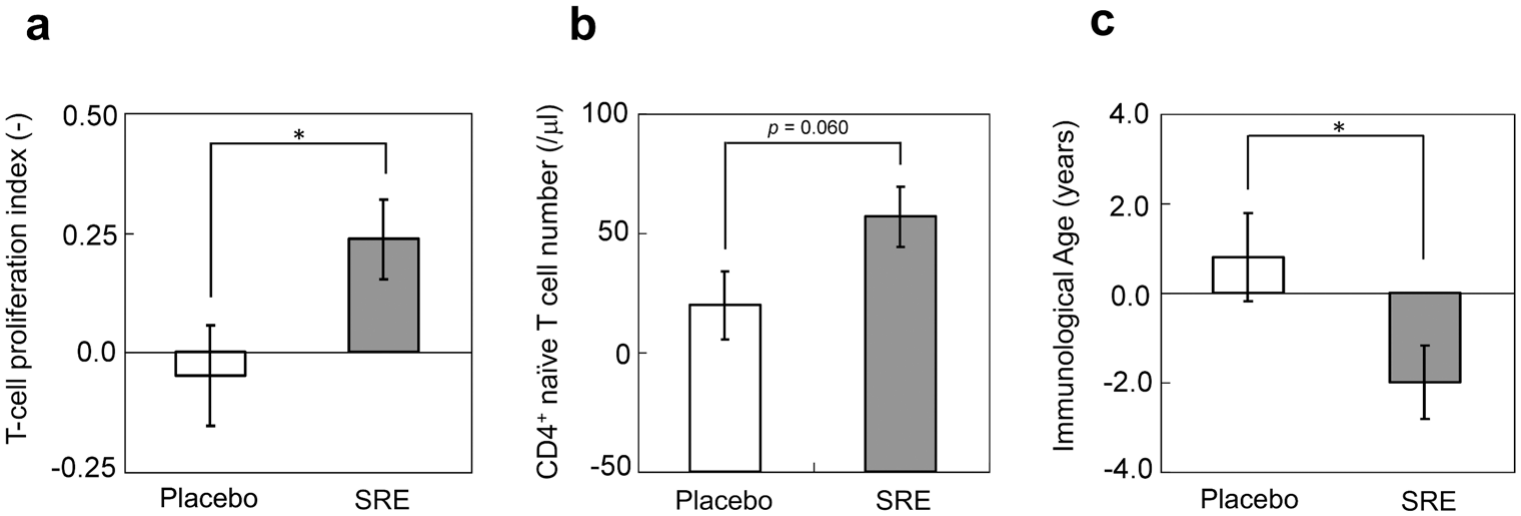
Changes in the immunological index after *Salacia reticulata* extract (SRE) ingestion. (**a**) Changes in the T-cell proliferation index from the initial values to the values after 4 weeks are shown in the plot. Although the index decreased by 0.05 ± 0.10 in the P group, it increased by 0.24 ± 0.08 in the SRE group (*p* < 0.05 for intergroup comparison). The values represent the mean ± standard deviation. (**b**) Changes in the CD4^+^ naïve T-cell count from the initial values to the values after 4 weeks are shown in the plot. The cell count increased by 19.67 ± 14.26/μl in the P group, whereas it increased by 56.80 ± 12.50/μl in the SRE group (*p* < 0.0602 for intergroup comparison). (**c**) Changes in the immunological age from the initial values to the values after 4 weeks are shown in the plot. The age increased by 0.80 ± 0.99 years in the P group, whereas it decreased by 2.00 ± 0.81 years in the SRE group (*p* < 0.05 for intergroup comparison). *p < 0.05.

Immunological tests revealed that the T-cell proliferation index increased from a pre-ingestion value of 1.44 ± 0.14 to a post-ingestion value of 1.68 ± 0.17 in the SRE group (*p* < 0.0133); in contrast, in the P group, the values were 1.58 ± 0.14 and 1.53 ± 0.11, respectively. The change in the T-cell proliferation index from the initial values after 4 weeks was an increase of 0.24 ± 0.08 in the SRE group and a decrease of 0.05 ± 0.10 in the P group, which showed a statistically significant difference between the groups (*p* < 0.0426; Fig 3a).

The CD4^+^ naïve T-cell count increased from 245 ± 34/μl before ingestion to 301 ± 42/μl after ingestion in the SRE group (*p* < 0.0005). In the P group, the pre- and post-ingestion values were 267 ± 33/μl and 287 ± 29/μl, respectively, but the change was not statistically significant. The change from the initial value was an increase of 56.80 ± 12.50/μl in the SRE group and 19.67 ± 14.26/μl in the P group, showing a trend toward a significant difference between the two groups (*p* < 0.0602; Fig 3b).

Immunological age is a new parameter that is calculated by the inverse correlation between the T-cell proliferation index and age (for more details, refer to Materials and Methods). In the SRE group, the immunological age was 60.1 ± 1.6 years before ingestion, and it significantly decreased to 58.1 ± 3.9 years after ingestion (*p* < 0.0271) [29]. In contrast, the pre- and post-ingestion immunological ages in the P group were 59.0 ± 1.5 and 59.8 ± 1.3 years, respectively, and no significant difference was observed. The change from the pre-ingestion immunological age was a decrease of 2.00 ± 0.81 years in the SRE group and an increase of 0.80 ± 0.99 years in the P group, which showed a significant difference in the immunological age change between the two groups (*p* < 0.0373; Fig 3c). Cytokine production levels (IL-1β, IL-2, IL-4, IL-5, IL-8, IL-10, IL-12p70, and IL-17A; IFN-γ, TNF-α, and TNF-β) in lymphocyte culture did not show a significant change except for IL-6. IL-6 production levels showed a tendency to decrease in the SRE group (*p* < 0.0566) but not in the P group. There was a trend toward a significant difference between the groups (*p* < 0.0792; S1 Fig).

### Intestinal microbiota analysis

**Fig 4:**
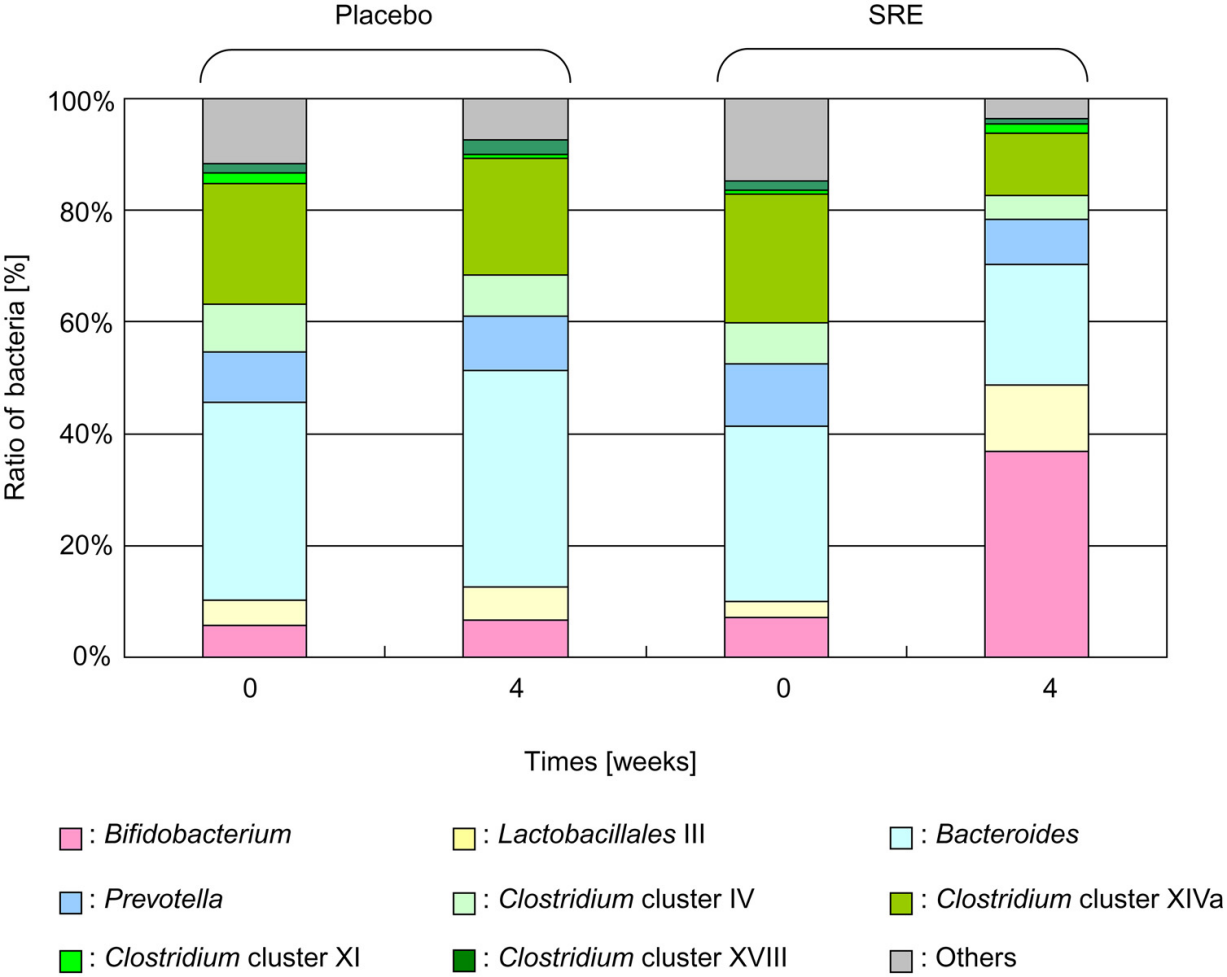
Changes in the intestinal microbiota after *Salacia reticulata* extract (SRE) ingestion. Comparison of the changes in the proportion of bacteria in the two groups. In intergroup comparisons, significant increases in *Bifidobacterium* (*p* < 0.0002) and marked decreases in *Bacteroides* (*p* < 0.0320) and *Clostridium* (*Clostridium* cluster IV, *Clostridium* subcluster XIVa, *Clostridium* cluster XI, and *Clostridium* cluster XVIII; *p* < 0.0114) were observed. *Lactobacillales* showed a trend toward increasing (*p* < 0.0742).

The T-RFLP method could detect bacterial composition ratios using the 16S rRNA gene of bacteria (Fig 4) that are usually difficult to culture. The results indicated marked changes in the intestinal microbiota composition. As a typical example, in the SRE group, *Bifidobacterium* (OTU: 124) accounted for 7.1% ± 2.4% of the intestinal microbiota before ingestion and 36.2% ± 6.1% after ingestion, which showed a substantial increase (*p* < 0.0006). In the P group, *Bifidobacterium* accounted for 5.7% ± 1.1% of the intestinal microbiota before ingestion and 6.6% ± 1.1% after ingestion, which showed no significant change. Comparison of the changes in bacterial proportions between the two groups confirmed a significant increase in the SRE group (*p* < 0.0027). The proportion of bacteria of the *Lactobacillales* order (OTU: 332, 520, and 657), which accounted for 2.9% ± 0.7% of the intestinal microbiota before SRE ingestion, also significantly increased to 11.7% ± 3.3% after ingestion (*p* < 0.0043), whereas no significant change was observed in the P group (4.6% ± 2.6% before ingestion and 5.9% ± 3.2% after ingestion). Comparison between groups confirmed that *Lactobacillales* bacteria showed a significant tendency toward increases in intestinal microbiota by SRE ingestion (*p* < 0.0742).

In contrast, *Clostridium* (OTU: 106, 168, 338, 369, 423, 494, 505, 517, 650, 749, 754, 955, 990; *Clostridium* cluster IV, *Clostridium* subcluster XIVa, *Clostridium* cluster XI, and *Clostridium* cluster XVIII) accounted for 32.7% ± 2.6% before SRE ingestion and 17.6% ± 2.7% after SRE ingestion (*p* < 0.0033). In the P group, the proportion did not change following ingestion (33.6% ± 3.4% before ingestion and 31.7% ± 3.4% after ingestion). Comparison of the proportion of *Clostridium* between the two groups confirmed a significant decrease in the SRE group (*p* < 0.0114). In this group, the proportion of *Bacteroides* decreased following ingestion (from 31.3% ± 3.6% to 21.2% ± 4.0%; *p* < 0.0777). In the P group, the proportion before and after ingestion was 35.4% ± 4.04% and 38.7% ± 4.0%, respectively, which showed no significant change. Intergroup comparison indicated that the proportion of *Bacteroides* was reduced by SRE ingestion (*p* < 0.0320).

## Discussion

In this study, experiments were performed to investigate the effects of SRE powder on gene expression of peripheral blood cells, immunological parameters of peripheral blood lymphocytes, and intestinal microbiota in healthy adults with mildly reduced immunity.

Gene analysis of peripheral blood cells showed that expressions of IFN-induced genes were upregulated, while those of inflammation-related genes, except for IFN-related genes, were downregulated. These findings are consistent with immunological changes and show an increase in the T-cell proliferation index and number of CD4^+^ naïve T cells and a trend toward reduction of IL-6 production. Proliferation activity is an essential function of T cells when they encounter certain pathogens. Because the number of CD4^+^ naïve T cells usually decreases with age, their increase suggests a trend toward a younger phenotype of the SRE powder. Actually, immunological age calculated by the T-cell proliferation index was lower in the SRE group than in the P group.

In addition, a prominent change was observed in the composition of intestinal microbiota, i.e., an increase in *Bifidobacterium* and *Lactobacillales* together with a decrease in *Clostridium* groups. Although a direct explanatory relationship between the immune system and microbiota remains unknown, the intestinal condition clearly changed in the SRE group relative to that in the P group. With regard to sex differences, we confirmed in a preliminary study that changes in the intestinal microbiota also occur in women, and it is highly likely that SRE exerts a similar effect in women as well.

*Bifidobacteria* that increased by SRE ingestion are known to decrease with age [31]. Different bacteria assimilate different types of saccharides. Our study showed that a wide variety of food-derived oligosaccharides were formed in the intestine by ingestion of SRE that had α-glucosidase inhibitory activity and suppressed carbohydrate absorption as well as by the resulting increase in bacteria, which produced an immunological effect in the body [18]. Furthermore, gene expression analysis revealed that Tlr5 genes involved in the recognition of flagellin (a component of flagellate bacteria, such as *Bifidobacteria*) were upregulated. Stimulated Tlr5 is known to induce Th1 and Th17 [32, 33]. In this study, Th1-related genes for IFN signaling were upregulated; however, Th17-related genes for Jak1 and Rora were downregulated, with a decrease in IL-6 production. Therefore, SRE ingestion possibly induces Th1, specifically via Tlr5. As a result of Th1 induction, disordered functions caused by excessive inflammatory events, such as aging and allergy, have inhibitory potential.

## Conclusions

In conclusion, the present study provided evidence that SRE could change the gene expression of peripheral blood cells as well as the proportion of intestinal microbiota together with a shift toward younger phenotype. A human ingestion test also showed that a food material enhanced immunity that had decreased with age. These findings verify those obtained in previous basic animal studies that analyzed both mechanisms using transcriptome and changes in the intestinal microbiota. In the experiments, consistent results were obtained from basic studies through to the human test. We plan to analyze the effect of each component within SRE and to study the effect of SRE, which prevents disease associated with dysbiosis and immunological deterioration. The utility of a food has rarely been evaluated by such multifaceted experiments, and the present study presents a milestone with regard to the methodology used. We believe that the results of this study will advance research on functional foods.

## Supporting Information

S1 CONSORT Checklist CONSORT 2010 Checklist. (PDF)

S1 Fig Changes in IL-6 production after SRE ingestion. (PDF)

S1 Protocol Trial Protocol. (PDF)

S1 Table GO terms with Benjamini and Hochberg FDR-corrected *p*-value of <0.01. (PDF)

S2 Table Canonical pathway. (PDF)

S3 Table Up-regulated genes. (PDF)

S4 Table Down-regulated genes. (PDF)
