# Supplementary material for: Improvement in Human Immune Function with Changes in Intestinal Microbiota by Salacia reticulata Extract Ingestion: A Randomized Placebo-Controlled Trial
Source: PLoS One. 2015 Dec 2;10(12):e0142909. doi: 10.1371/journal.pone.0142909 (PMC4667990; doi:10.1371/journal.pone.0142909)
Supplement: S1 Fig — (PDF) [file pone.0142909.s002.pdf]

Supplementary Data

Improvement in human immune function with changes in intestinal microbiota by *Salacia reticulata* extract ingestion

Yuriko Oda, Fumitaka Ueda, Masanori Utsuyama, Asuka Kamei, Chihaya Kakinuma, Keiko Abe, and Katsuiku Hirokawa

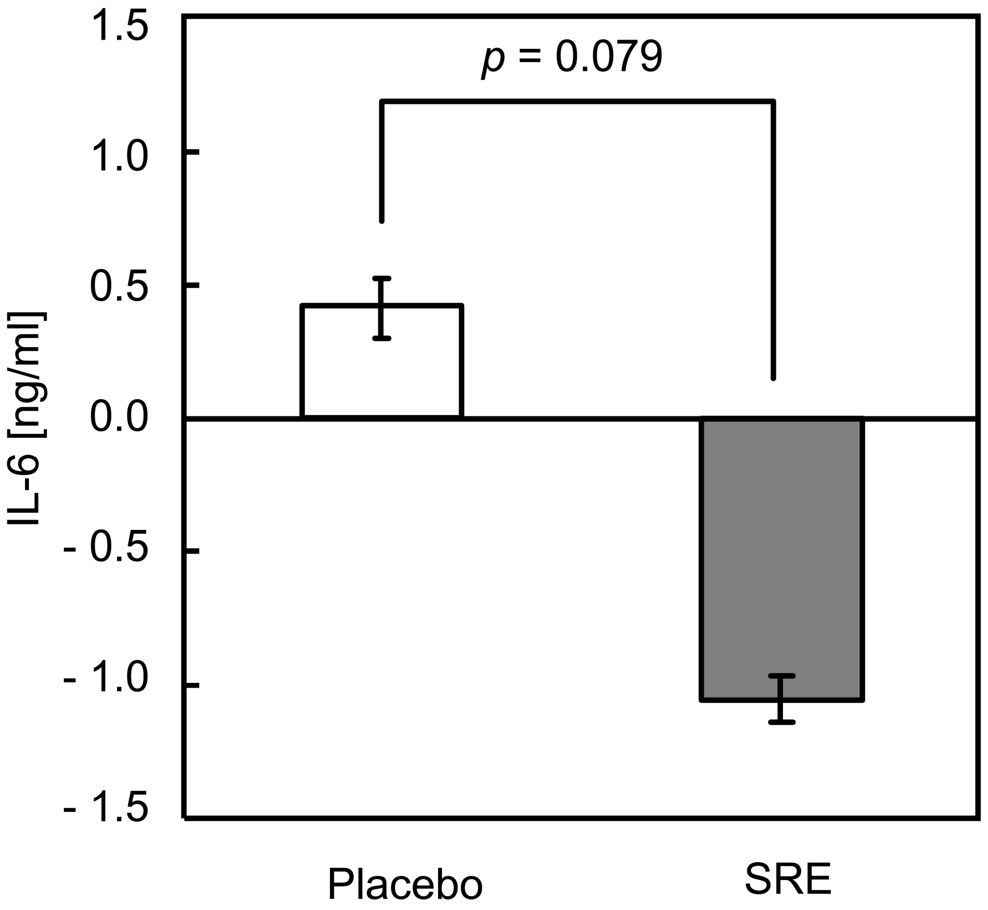

Supplementary Figure 1 Changes in IL-6 production after SRE ingestion

The IL-6 production level showed a tendency to decrease in both pre-ingestion versus post-ingestion and inter-group comparisons.
