# Supplementary material for: Improvement in Human Immune Function with Changes in Intestinal Microbiota by Salacia reticulata Extract Ingestion: A Randomized Placebo-Controlled Trial
Source: PLoS One. 2015 Dec 2;10(12):e0142909. doi: 10.1371/journal.pone.0142909 (PMC4667990; doi:10.1371/journal.pone.0142909)
Supplement: S1 Protocol — (PDF) [file pone.0142909.s003.pdf]

SOUKEN

Study Title

Evaluation of immunity enhancement by ingestion of a Salacia plant extract-containing supplement

Study number	:	FJFR-13916
Study period	:	September 17, 2013 – October 29, 2013

[Draft history]

July 29, 2013	Draft protocol	Version 1 prepared

Unauthorized use prohibited

This protocol is an intellectual property jointly created and owned by FUJIFILM Corporation and SOUKEN Co., Ltd. Therefore, no one is allowed to use any information related to this protocol for diversion or other purposes without permission of the both companies.

Table of Contents

1. Study Outline 1
2. Study Title and Study Number 3
3. Study Organization 3
4. Background 4
5. Objective 4
6. Subjects 4
7. Consent from Subjects 5
8. Outline of the Test Products 5
9. Study Methods 6
10. Endpoints (various tests, measurement items, and survey methods) 7
11. Test Product Management 7
12. Subject Management Matters 7
13. Statistical Analysis 8
14. Final Report 8
15. Criteria for discontinuation of the study and drop-out cases 8
16. Compensation to Subjects 8
17. Criteria for Exclusion from Analysis (Reporting) Set 9
18. Note on Completing the Survey Form 9
19. Modification/Absence of Test Data 9
20. Protection of Subjects' Privacy 9
21. Ethics 9
22. Attachments 9

1. Study Outline

Title	Evaluation of immunity enhancement by ingestion of a Salacia plant extract-containing supplement
Name of products tested	Salacia-containing supplement and placebo
Objective	The objective is to determine effects of a "Salacia plant extract-containing supplement" on immunological functions in adult males who were conscious of being prone to tiredness and ingested the supplement for 4 weeks. A placebo-controlled, double-blind study is conducted to evaluate the immunity-enhancing effect of the Salacia plant extract-containing supplement ingested, and blood pressure/pulse measurement, blood testing, immunity measurement, cytokine production measurement, blood gene expression analysis, blood bile acid measurement, intestinal flora analysis, and a questionnaire survey are conducted.
Subjects	Number of subjects: 30 (Group 1: 15, Group 2: 15)
Entry criteria (based on subjects' self-reporting)	1) Japanese males who are at least 50 years and less than 60 years of age at the time of consenting<br>※ "Japanese" herein refers to individuals with Japanese genetic background, regardless of Japanese citizenship.<br>2) Individuals who have a feeling of tiredness on a daily basis and a feeling of not recovering from tiredness readily.<br>3) Day-shift, sedentary workers<br>4) Individuals with a regular life rhythm
Screening criteria	1) Selection is made by the sponsor (immunological scores, etc.)
Screening items	1) Body height measurement<br>2) Body structure measurement (body measurement)<br>3) Blood pressure/pulse measurement<br>4) Blood test<br>5) Blood sample submission<br>6) Fecal sample submission
Exclusion criteria	1) Individuals who have ever medically treated for a heart disease, a renal disorder, a hepatic disorder, a gastrointestinal disease, or some other conditions.<br>2) Exclusion for diseases, etc. [individuals being treated for atrial fibrillation, arrhythmia, hepatic disorder (especially history or current illness of hepatitis), renal disorder, cerebrovascular disorder, rheumatism, diabetes, dyslipidemia, hypertension, and other chronic diseases<br>3) Individuals with a gastrointestinal disease (such as extreme constipation and frequent diarrhea)<br>4) Individuals with severe anemia<br>5) Individuals taking an medicine (including kampo formulations) or a supplement on a regular basis<br>6) Individuals who are allergic (to test food-related food/drug)<br>7) Individuals taking a food/drug with a digestive conditioning effect on a daily basis over the last one month<br>8) Individuals consuming alcohol excessively<br>9) Smokers<br>10) Individuals with diabetes<br>11) Individuals who have participated within the last 3 months or is currently participating in any other clinical study<br>12) Individuals with any abnormality found in the screening study<br>13) Individuals determined to be ineligible by the Principal Investigator
Subject allocation method	Subjects shall be grouped in a manner such that the resulting groups are as close as possible to each other in the mean immunological score.
Study design	Placebo-controlled, double-blind

Observation time points	Before ingestion, after 4 weeks of ingestion
Number of observation	2 times
Method/amount of ingestion	Method of ingestion: See main text.<br>Amount of ingestion at a time: See main text.
Endpoints	・Body structure measurement (body measurement)<br>・Blood pressure/pulse measurement<br>・Blood test<br>・Blood sample submission<br>・Fecal sample submission<br>・Questionnaire survey
Statistical treatment	1. For items/methods for statistical analysis, see Attachment 1.<br>2. The significance level shall be 5% by a two-tailed test.
Reporting method	A report shall be prepared based on the data tabulated and statistically analyzed at the Institution (Data attachment to the study report).
Institution	SOUKEN Co., Ltd. (Clinical Study Office)<br>1-9-10 Hamamatsu-cho, DaiwaA Hamamatsu-cho Building 6F, Minato-ku, Tokyo 105-0013<br>TEL：03-5408-1557／FAX：03-5408-1556
Principal investigator	Takashi Koikeda, Director, Shiba Palace Clinic<br>1-9-10 Hamamatsu-cho, DaiwaA Hamamatsu-cho Building 6F, Minato-ku, Tokyo 105-0013<br>TEL：03-5408-1599／FAX：03-5408-0059
Sponsor	Fujifilm Corporation<br>577 Ushijima, Kaiseimachi, Ashigarakamigun, Kanagawa 258-8577<br>TEL：0465-86-1159／FAX：0465-86-1019<br>Person-in-charge: Yuriko Oda
Ethical considerations	This study shall be conducted in accordance with the Declaration of Helsinki (revised at the Seoul general assembly in 2008), with due ethical considerations.

2. Study Title and Study Number

Study title: Evaluation of immunity enhancement by ingestion of a Salacia plant extract-containing supplement

Study number: FJFR-13916

3. Study Organization

1） Sponsor

Fujifilm Corporation
577 Ushijima, Kaiseimachi, Ashigarakamigun, Kanagawa 258-8577
TEL：0465-86-1159／FAX：0465-86-1019
Person-in-charge: Yuriko Oda

2） Entrustee

SOUKEN Co., Ltd. (Secretariat)
1-9-10 Hamamatsu-cho, DaiwaA Hamamatsu-cho Building 6F, Minato-ku, Tokyo 105-0013
TEL：03-5408-1555／FAX：03-5408-1556
Person-in-charge: Yasushi Masuda

3） Principal investigator

Takashi Koikeda, Director, Shiba Palace Clinic
1-9-10 Hamamatsu-cho, DaiwaA Hamamatsu-cho Building 6F, Minato-ku, Tokyo 105-0013
TEL：03-5408-1599／FAX：03-5408-0059

4） Institution

SOUKEN Co., Ltd. (Clinical Study Office)
1-9-10 Hamamatsu-cho, DaiwaA Hamamatsu-cho Building 6F, Minato-ku, Tokyo 105-0013
TEL：03-5408-1557／FAX：03-5408-1556

5） Investigators

In charge of Secretariat Yasushi Masuda SOUKEN Co., Ltd.
Analysis Division Masahiro Yagibashi Same as above
Academic Division Soichiro Matsuse Same as above

6） Consultation service

Shiba Palace Clinic
1-9-10 Hamamatsu-cho, DaiwaA Hamamatsu-cho Building 6F, Minato-ku, Tokyo 105-0013
TEL：03-5408-1599／FAX：03-5408-0059
Director, Takashi Koikeda

7） Blood/urine tests subcontractor

BML Inc.
1-34-5 Koenjiminami, Suginami-ku, Tokyo 166-0003
The Second Tokyo Sales Office, Tokyo Sales Division, Clinical Laboratory Sales Department I
TEL：03-3316-0111／FAX：03-3316-4429

4. Background

Plants of the genus Salacia have been used for generations as Ayurvedic medicinal plants for prevention of diabetes and obesity. Moreover, recent studies have shown that Salacia plants extracts improved effects on intestinal environment/flora, in addition to the already known improving effects on the blood sugar level and obesity.

In addition, various immunological effects have been demonstrated in animals that received Salacia plant extracts, including enhanced expression of Th1 cell-associated genes, increased growth of intestinal bacteria considered to be involved in immunity, and symptomatic improvement of influenza infection, and immunostimulatory effects of Salacia plant extracts are also expected for humans.

However, effects of Salacia plant extracts on human immune functions have not been determined in any previous studies, and remain to be studied.

5. Objective

The objective is to determine effects of a "Salacia plant extract-containing supplement" on immunological functions in adult males who were conscious of being prone to tiredness and ingested the supplement for 4 weeks. A placebo-controlled, double-blind study is conducted to evaluate the immunity-enhancing effect of the Salacia plant extract-containing supplement ingested, and blood pressure/pulse measurement, blood testing, immunity measurement, cytokine production measurement, blood gene expression analysis, blood bile acid measurement, intestinal flora analysis, and a questionnaire survey are conducted.

6. Subjects

※ "Japanese" herein refers to individuals with Japanese genetic background, regardless of Japanese citizenship.

Of SOUKEN registrants who have undergone a questionnaire survey at the time of recruitment, those who self-report to meet the following "Entry criteria (based on subjects' self-reporting)" and not to meet the "Exclusion criteria" are subjected to a screening survey, and 30 of those who meet the "Screening criteria" and were considered appropriate to participate in this study are selected as subjects in this study.

Even if test results, etc. of any selected subject are found contradictory to those self-reported earlier ("Entry criteria (based on subjects' self-reporting)" "Exclusion criteria"), the subject shall be deemed to be approved to participate in the study as long as the subject has not completed the screening survey, and shall not be excluded from the analysis set unless there is a special reason to do so.

1） Entry criteria (based on subjects' self-reporting)

1) Japanese males who are at least 50 years and less than 60 years of age at the time of consenting

※ "Japanese" herein refers to individuals with Japanese genetic background, regardless of Japanese citizenship.

2) Individuals who have a feeling of tiredness on a daily basis and a feeling of not recovering from tiredness readily.
3) Day-shift, sedentary workers
4) Individuals with a regular life rhythm

2） Screening criteria

1) Selection is made by the sponsor (immunological scores, etc.)

3） Screening items

1) Body height measurement
2) Body structure measurement (body measurement)
3) Blood pressure/pulse measurement
4) Blood test
5) Blood sample submission
6) Fecal sample submission

4） Exclusion criteria

1) Individuals who have ever medically treated for a heart disease, a renal disorder, a hepatic disorder, a gastrointestinal disease, or some other conditions.
2) Exclusion for diseases, etc. [individuals being treated for atrial fibrillation, arrhythmia, hepatic disorder (especially history or current illness of hepatitis), renal disorder, cerebrovascular disorder, rheumatism, diabetes, dyslipidemia, hypertension, and other chronic diseases
3) Individuals with a gastrointestinal disease (such as extreme constipation and frequent diarrhea)
4) Individuals with severe anemia
5) Individuals taking an medicine (including kampo formulations) or a supplement on a regular basis
6) Individuals who are allergic (to test food-related food/drug)
7) Individuals taking a food/drug with a digestive conditioning effect on a daily basis over the last one month
8) Individuals consuming alcohol excessively
9) Smokers
10) Individuals with diabetes
11) Individuals who have participated within the last 3 months or is currently participating in any other clinical study
12) Individuals with any abnormality found in the screening study
13) Individuals determined to be ineligible by the Principal Investigator

7. Consent from Subjects

①Objective and methods of this study
②Explanation about the test products, their effects, and adverse effects predicted to occur
③Subjects are kept under adequate control by the Principal Investigator during the study.
④Subjects are free from any penalty even if they do not consent to participate in the study.
⑤Even after formally consenting to participate in the study, subjects can withdraw it any time.
⑥Appropriate procedures and therapies that subjects are entitled to receive in case of a health damage related to this study
⑦Subjects shall receive information that potentially effects subjects' intention for continued participation in this study as soon as such information is obtained.
⑧Other necessary matters concerning protection of subjects' human rights and disclosure of subjects' information
⑨What subjects should do and do not
⑩Establishment of the consultation service at the institution that subjects should contact for more information about this study and subjects' rights or in case of health damages related to this study
⑪About cooperation expenditures to be paid to subjects

8. Outline of the Test Products

Test products are those supplied by Fujifilm Corporation (a Salacia-containing supplement and a placebo).

1) Origin and the course of development

The functional food "Metabarrier", which has been released in 2008 from Fujifilm Corporation, contains useful components such as green tea extracts and red wine polyphenols as well as the Salacia plant extract. A test food comprising the Salacia plant extract as the only useful component was prepared to clarify functions of the Salacia plant extract alone.

2) Safety/efficacy data

The test product used in this study is prepared from commercially available Metabarrier by removing some components therein, and safety/efficacy information available for Metabarrier may be used as a guide.

No serious adverse event was reported in the results of the human use study conducted by SOUKEN before Metabarrier was launched. Furthermore, no serious health damage has been

reported to be caused by Metabarrier components since its release in 2008 through to now. Therefore, the safety of the test product appears to be sufficient.
In addition, the test product is considered to have a mild effect of reducing intestinal sugar absorption via inhibition of the α-glucosidase activity, since it contains the Salacia plant extract as does Metabarrier.

3) Amount to be ingested and underlying rationale

The amount of the Salacia plant extract in commercially available Metabarrier is 240 mg as a daily intake. In this study, the test product is formulated in tablets containing the Salacia plant extract in the amount corresponding to that ingested daily as Metabarrier. It should be noted that up to 1000 mg/day can be ingested safely according to the guidelines issued by the Ministry of Health, Labour and Welfare.

4) Method of ingestion of the test products

Test product name	Time of the day	Time	Amount to be ingested	Method of ingestion
Salacia plant extract-containing tablets	Morning, midday, evening	Before every meal	1 tablet before breakfast, 1 tablet before lunch, 2 tablets before dinner	Tablets shall be ingested with water or plain hot water before meals.
Placebo tablets	Morning, midday, evening	Before every meal	1 tablet before breakfast, 1 tablet before lunch, 2 tablets before dinner	Tablets shall be ingested with water or plain hot water before meals.

5) Storage method and expiration date

Test product name	Form	Taste/smell	Storage method	Expiration date
Salacia plant extract-containing tablets	Tablet	Slight bitterness, woody aroma	Store at normal temperature away from direct sunlight, high temperature and high humidity	1 year
Placebo tablets	Tablet	Slight bitterness, odorless	Store at normal temperature away from direct sunlight, high temperature and high humidity	1 year

6) Ingredients of the test products and their contents

Salacia plant extract-containing tablets	Content
Salacia extract powder	60.00 mg
Calcium carbonate	6.00 mg
Crystalline cellulose	172.75 mg
Sucrose fatty acid esters	7.50 mg
Particulate silicon dioxide	3.75 mg
Placebo tablets	Content
Crystalline cellulose	175.00 mg
Erythritol	50.00 mg
Particulate silicon dioxide	2.50 mg
Calcium stearate	2.50 mg
Color adjusting agent	20.00 mg

9. Study Methods

1) Study design

Placebo-controlled, double-blind

2) **Study schedule**

The study shall be conducted in the following schedule.

・Number of observation: 2

・Observation time points: Before ingestion, after 4 weeks of ingestion

・Ingestion period: 28 days

①Acquisition of written consent

②Main study

[Number of subjects in the main study] Number of subject: 30 (Group 1: 15, Group 2: 15)

※ For items to be carried out in the main study, see Attachment 1.

3) **Subject allocation**

Subjects shall be grouped in a manner such that the resulting groups are as close as possible to each other in the mean immunological score.

10. Endpoints (various tests, measurement items, and survey methods)

Items set forth in Attachment 1 are performed.

11. Test Product Management

1) **Persons in charge of the test products**

Fujifilm Corporation Yuriko Oda

SOUKEN Co., Ltd. Kohei Miyawaki

2) **Test product delivery/retrieval**

Test product name	Delivery date	Subject return	Return to Sponsor	Date to return	Return to:
Salacia plant extract-containing tablets	Friday, September 20, 2013	Required	Required	Friday, November 1, 2013	Fujifilm Corporation Oda
Placebo tablets	Friday, September 20, 2013	Required	Required	Friday, November 1, 2013	Fujifilm Corporation Oda

3) **Test product management and storage**

Test products are appropriately stored by the Institution (SOUKEN Co., Ltd.).

12. Subject Management Matters

①Subjects undergo fasting for at least 6 hours before the time of visit. (Example: Subjects scheduled to visit the clinic at 7 am may not eat after 4 am on the day) Water (mineral water) should be taken sufficiently. Subjects are not allowed to consume alcohol and should avoid excessive exercise on the day before a test.

②During the study, subjects are not allowed to take any supplement, except the test product and control product, stated to have immunological/digestive conditioning effects, or food containing an ingredient with the digestive conditioning effect (e.g., Easyfiber/Weider jelly fiber-in/Allbran/Oligo-no-okage/Calpis and the like/Kirin Supli/Kore-cut/Fibe-mini/Any green juice with FSHU stamp/Nisshin oishisa plus psyllium series/apple-flavored black vinegar/Lactic acid bacteria beverage (Yakult; Pirukuru; Mirumiru; Joa; Laurie Ace; etc.)/Yogurt; Yogurt drink (R-1; LG21; Bifidus; Meiji Bulgaria yogurt series; Soful; etc.)).

③During the study (from 1 week before the screening day to the test day after 4-week ingestion), subjects should avoid irregular ways of living (such as lack of sleep, over-eating/drinking). For

diet and exercise, subjects should maintain the same quantity and quality as in daily lives before the start of the study.

④During the study, drugs considered to affect test results (such as laxatives, intestinal remedies) are prohibited in principle. Record it in web diary if any of these must be taken.

⑤During the ingestion period, subjects are required to record whether the test food is ingested in the web diary specified by the Institution every day.

⑥In case of any change in physical conditions during the study, contact the entity managing the study immediately, and follow its instructions on subsequent necessary actions.

※ Although the items listed above on subject management must be complied in principle, they do not apply in cases medically required to do otherwise or other cases in which risks to the body or life are foreseen. If any of the items listed above is violated, the subject should contact the Institution promptly.

13. Statistical Analysis

①The significance level shall be 5% by a two-tailed test.

②For items/methods for statistical analysis, see Attachment 1.

14. Final Report

1) A report shall be prepared based on the data tabulated and statistically analyzed at the Institution (Data attachment to the study report).Schedule until the report is prepared (plan)

Work is done according to the following schedule. ※ However, if it takes more than 7 days to obtain a test result, the subsequent schedule is postponed for a considerable period of time (on the following day if it is a holiday or in weekend).

Expected submission date (only business days are counted)	Data to be submitted
Within 10 days after the end of the study	Case conference
10 days after the end of the study	Submission of a draft data attachment to the study report
Within 14 days after submission of a draft data attachment to the study report	The data attachment to the study report to be finalized
25 days after the end of the study	Key open
Within 14 days after the data attachment to the study report has been finalized	Submission of a draft study report
Within 14 days after submission of a draft study report	The study report to be finalized

15. Criteria for discontinuation of the study and drop-out cases

In case of any of the following, the study is terminated on the basis of Principal Investigator's medical and ethical judgment. Except for some special circumstances, appropriate medical care is provided to subjects to ensure the safety of the subjects.

①When a serious adverse reaction, subjective/objective symptom, etc. has emerged

②When the subject is difficult to continue the study because of complication with any other disease or progression of a complication

③When tests are extremely difficult to perform

④When the whole study is discontinued

⑤When the Principal Investigator finds the study necessary to be discontinued

16. Compensation to Subjects

If a damage to a subject has occurred due to the study during the study, or if a subject has filed a law suit seeking compensation for a damage caused by this study, the Principal Investigator shall promptly

inform the Sponsor of it.

The Institution shall be responsible for compensations for health damages caused intentionally or negligently by the Institution, while the sponsor shall be entirely responsible for compensations in cases where health damages have occurred due to the test products. However, this does not apply if the health damage was falsely reported or intentionally caused by the subject.

17. Criteria for Exclusion from Analysis (Reporting) Set

Subjects who meet any of the following criteria are discussed in the case conference and shall be excluded from the analysis (reporting) set unless there is a special reason to do otherwise.

①Subjects found to have substantially deviated from instructions pertinent to subject management matters during the study.
②Subjects with data of which reliability is compromised in a major manner due to, for example, troubles in testing
③Subjects who have missed ingestion (the amount ingested does not meet the daily dose) in more than 15% of the days scheduled for ingestion
④Subjects found not to meet entry criteria and to meet exclusion criteria
⑤Subjects found appropriate to treat as drop-out cases for evident reasons

18. Note on Completing the Survey Form

A black-ink ballpoint pen shall be used to complete the survey form, and corrections shall be made with double lines.

19. Modification/Absence of Test Data

If a delay or cancellation of measurement is unavoidable for subject's health conditions or request, subject's health conditions and request shall be given priority in accordance with the spirit of the Declaration of Helsinki. If some data are impossible to collect for these reasons or other unavoidable reasons, they shall be treated as missing data.

20. Protection of Subjects' Privacy

All personnel involved in this study shall give full considerations to handling of information that can be used to identify individuals. In other records obtained in the course of this study, numbers shall replace names to prevent individuals from being identified. The test subcontractor is not allowed to obtain subjects' personal information under any circumstance.

21. Ethics

1） Compliance matters

This study shall be conducted in accordance with the Declaration of Helsinki (revised at the Seoul general assembly in 2008), with due ethical considerations.

2） Shiba Palace Clinic Ethical Review Board

Members of the Shiba Palace Clinic Ethical Review Board meet and discuss ethics and validity of the protocol for this study. The study must be conducted based on the protocol approved by the Shiba Palace Clinic Ethical Review Board. Any substantive deviation from the protocol must be approved by the Board.

22. Attachments

Attachment 1. Endpoints
Attachment 2. Blood/fecal endpoints

September 2, 2013

Sponsor: Fujifilm Corporation

577 Ushijima, Kaiseimachi, Ashigarakamigun, Kanagawa 258-8577

Pharmaceutical/Healthcare Research Laboratories Fumitaka Ueda

Entrustee: SOUKEN Co., Ltd.

1-9-10 Hamamatsu-cho, DaiwaA Hamamatsu-cho Building 6F, Minato-ku, Tokyo 105-0013

Representative director Kazuo Shirai

Entrustee: Shiba Palace Clinic

1-9-10 Hamamatsu-cho, DaiwaA Hamamatsu-cho Building 6F, Minato-ku, Tokyo 105-0013

Director Takashi Koikeda

Attachment 1. Endpoints

ID	Test	Method	Test description		S	Before ingestion	Four-week after ingestion
				Requirement for visit	○	─	○
0	Body height measurement	Description	Body height measurement. [Measurement items] Body height Total 1 item ※The last value is used when measured multiple times.	─	○	─	─
		Data processing	Tabulation only				
1	Body structure measurement (body measurement)	Measurement description	[Measurement items] Muscle mass/body fat amount/body weight/body fat percentage/BMI Total 5 items ※ (To be measured in a hospital gown) The last value is used when measured multiple times.	Interim report ○	○	─ (S value adopted)	○
		Instrument for measurement	InBody3.2/Biospace Co., Ltd.				
		Data processing	Tabulation/statistical analysis	Parametric			
		Statistical analysis	See below	Statistical analysis method			
		Comparison over time	Before ingestion vs 4-week after ingestion	Paired t test			
		Inter-group comparison	Between the groups before ingestion and 4-week after ingestion	Unpaired t test			
6	Blood pressure/pulse measurement	Measurement description	At least two measurements are made in a resting state. [Measurement sites] Left arm Total 1 site [Measurement items] Systolic blood pressure/diastolic blood pressure/pulse Total 3 items ※ The last values are used. ※ If blood pressure cannot be measured with an automated sphygmomanometer because, for example, the arm does not fit in the sphygmomanometer, a mercury sphygmomanometer (Kenzmedico Co., Ltd.) may be used.	Interim report ○	○	─ (S value adopted)	○
		Instrument for measurement	Automated blood pressure monitor Kentaro (HBP-9020)/Omron Healthcare Co., Ltd.				
		Data processing	Tabulation/statistical analysis	Parametric			
		Statistical analysis	See below	Statistical analysis method			
		Comparison over time	Before ingestion vs 4-week after ingestion	Paired t test			
		Inter-group comparison	Between the groups before ingestion and 4-week after ingestion	Unpaired t test			
9	Blood test	Test description	White blood cell image, peripheral blood general test, HbA1c:NGSP, total bilirubin, direct/indirect bilirubin, ZTT, AST (GOT), ALT (GPT), ALP, LDH, γ-GTP, cholinesterase, LAP, CK, total protein, creatinine , urea nitrogen, uric acid, total cholesterol, TG (triglycerides), calcium, serum iron, serum amylase, HDL-cholesterol, free fatty acids, glucose, LDL-cholesterol, glycated albumin, NK cell activity	Interim report ○	○	○	○
		Instrument for measurement	A designated blood testing company				
		Data processing	Tabulation/statistical analysis	Parametric			
		Statistical analysis	See below	Statistical analysis method			
		Comparison over time	Before ingestion vs 4-week after ingestion	Paired t test			
		Inter-group comparison	Between the groups before ingestion and 4-week after ingestion	Unpaired t test			
1040	Blood sample submission	Contents	See Attachment 2	Interim report ×	○	─	○
		Instrument for measurement	Shiba Palace Clinic				
		Data processing	Sampling/collection				
1066	Fecal sample submission	Contents	See Attachment 2	Interim report ×	○	─	○
		Instrument for measurement	Shiba Palace Clinic				
		Data processing	Sampling/collection				
9061	Questionnaire survey	Survey description	A questionnaire survey (6-point scale) on subjective symptoms is conducted.	Interim report ○	─	○	○
		Instrument for measurement	Self-administered by subjects				
		Data processing	Tabulation/statistical analysis	Non-parametric			
		Statistical analysis	See below	Statistical analysis method			
		Comparison over time	Before ingestion vs 4-week after ingestion	Wilcoxon's signed rank test			
		Inter-group comparison	Between the groups before ingestion and 4-week after ingestion	Mann-Whitney U test			

Attachment 2. Blood/fecal endpoin

ID	Test	Method	Test description		S	Before ingestion	After 4-week ingestion	Sample (storage method)	Testing institution
				Analytical method	○	—	○		
0	Immunity tests (Peace-of-mind course)	Measurement description	Immunity score, T lymphocyte age, immunological age, T cell count, CD4+/CD8+ T cell ratio, naive T cell count, naive/memory T cell ratio, B cell count, NK cell count, CD8+CD28+ T cell count, and T cell proliferation index are measured.		○	— (S value adopted)	○	Blood (shipping at normal temperature) ※10 ml whole blood	Institute for Health and Life Science Co., Ltd.
		Data processing	Tabulation/statistical analysis	Parametric					
		Statistical analysis	See below	Statistical analysis method					
		Comparison over time	Before ingestion vs 4-week after ingestion	Paired t test					
		Inter-group comparison	Between the groups before ingestion and 4-week after ingestion	Unpaired t test					
1	Treg cell counting	Measurement description	Treg cells are counted by flow cytometry.		○	— (S value adopted)	○	Blood (shipping at normal temperature)	Institute for Health and Life Science Co., Ltd.
		Data processing	Tabulation/statistical analysis	Parametric					
		Statistical analysis	See below	Statistical analysis method					
		Comparison over time	Before ingestion vs 4-week after ingestion	Paired t test					
		Inter-group comparison	Between the groups before ingestion and 4-week after ingestion	Unpaired t test					
2	Cytokine measurement	Measurement description	IL-1b, IL-2, IL-4, IL-5, IL-6, IL-8, IL-10, IL-12p70, IFNg, TNFa, TNFb, and IL-17 produced in the culture supernatant are measured (stimulus: PMA + Ionomysin).		○	— (S value adopted)	○	Blood (shipping at normal temperature)	Institute for Health and Life Science Co., Ltd.
		Data processing	Tabulation/statistical analysis	Parametric					
		Statistical analysis	See below	Statistical analysis method					
		Comparison over time	Before ingestion vs 4-week after ingestion	Paired t test					
		Inter-group comparison	Between the groups before ingestion and 4-week after ingestion	Unpaired t test					
3	Gene expression analysis	Measurement description	Microarray analysis of mRNA expression levels in blood		○	— (S value adopted)	○	Blood (stored in dedicated tubes; frozen after being let stand at normal temperature for 2 hours) ※2.5 ml whole blood × 2 tubes	Kurabo Industries Ltd., SRL Inc., KAST, Fujifilm
		Data processing	Tabulation/statistical analysis	Parametric					
		Statistical analysis	See below	Statistical analysis method					
		Comparison over time	Before ingestion vs 4-week after ingestion	Analysis is performed with the analysis software R.					
4	Bile acid measurement	Measurement description	Primary and secondary bile acids in the serum are measured.		○	— (S value adopted)	○	Serum (2.5 ml) is stored in a dedicated tube and frozen.	SRL Inc.
		Data processing	Tabulation/statistical analysis	Parametric					
		Statistical analysis	See below	Statistical analysis method					
		Comparison over time	Before ingestion vs 4-week after ingestion	Paired t test					
		Inter-group comparison	Between the groups before ingestion and 4-week after ingestion	Unpaired t test					
5	Intestinal flora	Measurement description	Intestinal flora in feces is measured with the T-RFLP (Nagashima) method.		○	— (S value adopted)	○	Feces (stored with a sampling kit and shipped)	TechnoSuruga Laboratory Co., Ltd.
		Data processing	Tabulation/statistical analysis	Parametric					
		Statistical analysis	See below	Statistical analysis method					
		Comparison over time	Before ingestion vs 4-week after ingestion	Paired t test					
		Inter-group comparison	Between the groups before ingestion and 4-week after ingestion	Unpaired t test					

[Criteria for exclusion from analysis set (reporting)] ※S = Screening

① If an observation is delayed for the predetermined number of days or more (before ingestion: no visit/ 4-week after ingestion: 7 days)

② If the ingestion is missed (the amount ingested does not meet daily dose) in more than 15% of the days scheduled for ingestion
