# Supplementary material for: Improvement in Human Immune Function with Changes in Intestinal Microbiota by Salacia reticulata Extract Ingestion: A Randomized Placebo-Controlled Trial
Source: PLoS One. 2015 Dec 2;10(12):e0142909. doi: 10.1371/journal.pone.0142909 (PMC4667990; doi:10.1371/journal.pone.0142909)
Supplement: S1 Table — (PDF) [file pone.0142909.s004.pdf]

Supplementary Data

Improvement in human immune function with changes in intestinal microbiota by *Salacia reticulata* extract ingestion

Yuriko Oda, Fumitaka Ueda, Masanori Utsuyama, Asuka Kamei, Chihaya Kakinuma, Keiko Abe, and Katsuiku Hirokawa

S1 Table. GO terms with Benjamini and Hochberg FDR-corrected *p*-value of <0.01

**Term**	**Count**	**Benjamini**
GO:0002376, immune system process	181	4.36E-27
GO:0006955, immune response	135	1.28E-22
GO:0045321, leukocyte activation	54	8.33E-10
GO:0006952, defense response	95	1.23E-08
GO:0001775, cell activation	57	1.54E-08
GO:0051707, response to other organism	56	6.11E-08
GO:0046649, lymphocyte activation	44	8.47E-08
GO:0009607, response to biotic stimulus	64	1.00E-06
GO:0006950, response to stress	189	1.88E-06
GO:0048518, positive regulation of biological process	219	2.30E-06
GO:0043067, regulation of programmed cell death	106	4.87E-06
GO:0042981, regulation of apoptosis	105	5.10E-06
GO:0010941, regulation of cell death	106	4.90E-06
GO:0006968, cellular defense response	20	1.24E-05
GO:0048522, positive regulation of cellular process	197	2.45E-05
GO:0051704, multi-organism process	90	2.72E-05
GO:0050896, response to stimulus	335	2.63E-05
GO:0042110, T cell activation	29	2.69E-05
GO:0002520, immune system development	47	3.89E-05
GO:0002682, regulation of immune system process	59	3.76E-05
GO:0048534, hemopoietic or lymphoid organ development	45	4.16E-05
GO:0009617, response to bacterium	37	4.34E-05
GO:0030097, hemopoiesis	42	4.72E-05
GO:0030098, lymphocyte differentiation	25	6.20E-05
GO:0006916, anti-apoptosis	38	6.99E-05
GO:0006954, inflammatory response	51	1.05E-04
GO:0030217, T cell differentiation	19	1.03E-04
GO:0006414, translational elongation	24	1.42E-04
GO:0043066, negative regulation of apoptosis	53	2.38E-04
GO:0002819, regulation of adaptive immune response	17	2.39E-04
GO:0043069, negative regulation of programmed cell death	53	3.39E-04
GO:0060548, negative regulation of cell death	53	3.56E-04
GO:0048872, homeostasis of number of cells	23	3.76E-04
GO:0002521, leukocyte differentiation	27	3.82E-04
GO:0009611, response to wounding	70	3.88E-04
GO:0002237, response to molecule of bacterial origin	21	3.85E-04
GO:0048523, negative regulation of cellular process	172	5.00E-04
GO:0048519, negative regulation of biological process	185	4.89E-04
GO:0007243, protein kinase cascade	53	6.53E-04

GO:0002822, regulation of adaptive immune response based on somatic recombination of immune receptors built from immunoglobulin superfamily domains	16	7.02E-04
GO:0032496, response to lipopolysaccharide	19	9.41E-04
GO:0009615, response to virus	23	0.00128
GO:0034097, response to cytokine stimulus	19	0.00131
GO:0002684, positive regulation of immune system process	38	0.00131
GO:0006915, apoptosis	73	0.00350
GO:0010033, response to organic substance	84	0.00362
GO:0016265, death	84	0.00412
GO:0050776, regulation of immune response	35	0.00513
GO:0008219, cell death	83	0.00515
GO:0012501, programmed cell death	73	0.00510
GO:0007259, JAK-STAT cascade	12	0.00631
GO:0006357, regulation of transcription from RNA polymerase II promoter	83	0.00704
GO:0009893, positive regulation of metabolic process	100	0.00879
GO:0043068, positive regulation of programmed cell death	55	0.00890
GO:0002252, immune effector process	24	0.00902
GO:0007242, intracellular signaling cascade	129	0.00966
GO:0010942, positive regulation of cell death	55	0.00949
GO:0031325, positive regulation of cellular metabolic process	96	0.00945
GO:0009605, response to external stimulus	99	0.00940

Supplementary Data

Improvement in human immune function with changes in intestinal microbiota by *Salacia reticulata* extract ingestion

Yuriko Oda, Fumitaka Ueda, Masanori Utsuyama, Asuka Kamei, Chihaya Kakinuma, Keiko Abe, and Katsuiku Hirokawa

S1 Table. GO terms with Benjamini and Hochberg FDR-corrected *p*-value of <0.01

**Term**	**Count**	**Benjamini**
GO:0002376, immune system process	181	4.36E-27
GO:0006955, immune response	135	1.28E-22
GO:0045321, leukocyte activation	54	8.33E-10
GO:0006952, defense response	95	1.23E-08
GO:0001775, cell activation	57	1.54E-08
GO:0051707, response to other organism	56	6.11E-08
GO:0046649, lymphocyte activation	44	8.47E-08
GO:0009607, response to biotic stimulus	64	1.00E-06
GO:0006950, response to stress	189	1.88E-06
GO:0048518, positive regulation of biological process	219	2.30E-06
GO:0043067, regulation of programmed cell death	106	4.87E-06
GO:0042981, regulation of apoptosis	105	5.10E-06
GO:0010941, regulation of cell death	106	4.90E-06
GO:0006968, cellular defense response	20	1.24E-05
GO:0048522, positive regulation of cellular process	197	2.45E-05
GO:0051704, multi-organism process	90	2.72E-05
GO:0050896, response to stimulus	335	2.63E-05
GO:0042110, T cell activation	29	2.69E-05
GO:0002520, immune system development	47	3.89E-05
GO:0002682, regulation of immune system process	59	3.76E-05
GO:0048534, hemopoietic or lymphoid organ development	45	4.16E-05
GO:0009617, response to bacterium	37	4.34E-05
GO:0030097, hemopoiesis	42	4.72E-05
GO:0030098, lymphocyte differentiation	25	6.20E-05
GO:0006916, anti-apoptosis	38	6.99E-05
GO:0006954, inflammatory response	51	1.05E-04
GO:0030217, T cell differentiation	19	1.03E-04
GO:0006414, translational elongation	24	1.42E-04
GO:0043066, negative regulation of apoptosis	53	2.38E-04
GO:0002819, regulation of adaptive immune response	17	2.39E-04
GO:0043069, negative regulation of programmed cell death	53	3.39E-04
GO:0060548, negative regulation of cell death	53	3.56E-04
GO:0048872, homeostasis of number of cells	23	3.76E-04
GO:0002521, leukocyte differentiation	27	3.82E-04
GO:0009611, response to wounding	70	3.88E-04
GO:0002237, response to molecule of bacterial origin	21	3.85E-04
GO:0048523, negative regulation of cellular process	172	5.00E-04
GO:0048519, negative regulation of biological process	185	4.89E-04
GO:0007243, protein kinase cascade	53	6.53E-04

GO:0002822, regulation of adaptive immune response based on somatic recombination of immune receptors built from immunoglobulin superfamily domains	16	7.02E-04
GO:0032496, response to lipopolysaccharide	19	9.41E-04
GO:0009615, response to virus	23	0.00128
GO:0034097, response to cytokine stimulus	19	0.00131
GO:0002684, positive regulation of immune system process	38	0.00131
GO:0006915, apoptosis	73	0.00350
GO:0010033, response to organic substance	84	0.00362
GO:0016265, death	84	0.00412
GO:0050776, regulation of immune response	35	0.00513
GO:0008219, cell death	83	0.00515
GO:0012501, programmed cell death	73	0.00510
GO:0007259, JAK-STAT cascade	12	0.00631
GO:0006357, regulation of transcription from RNA polymerase II promoter	83	0.00704
GO:0009893, positive regulation of metabolic process	100	0.00879
GO:0043068, positive regulation of programmed cell death	55	0.00890
GO:0002252, immune effector process	24	0.00902
GO:0007242, intracellular signaling cascade	129	0.00966
GO:0010942, positive regulation of cell death	55	0.00949
GO:0031325, positive regulation of cellular metabolic process	96	0.00945
GO:0009605, response to external stimulus	99	0.00940

Supplementary Data

Improvement in human immune function with changes in intestinal microbiota by *Salacia reticulata* extract ingestion

Yuriko Oda, Fumitaka Ueda, Masanori Utsuyama, Asuka Kamei, Chihaya Kakinuma, Keiko Abe, and Katsuiku Hirokawa

S1 Table. GO terms with Benjamini and Hochberg FDR-corrected *p*-value of <0.01

**Term**	**Count**	**Benjamini**
GO:0002376, immune system process	181	4.36E-27
GO:0006955, immune response	135	1.28E-22
GO:0045321, leukocyte activation	54	8.33E-10
GO:0006952, defense response	95	1.23E-08
GO:0001775, cell activation	57	1.54E-08
GO:0051707, response to other organism	56	6.11E-08
GO:0046649, lymphocyte activation	44	8.47E-08
GO:0009607, response to biotic stimulus	64	1.00E-06
GO:0006950, response to stress	189	1.88E-06
GO:0048518, positive regulation of biological process	219	2.30E-06
GO:0043067, regulation of programmed cell death	106	4.87E-06
GO:0042981, regulation of apoptosis	105	5.10E-06
GO:0010941, regulation of cell death	106	4.90E-06
GO:0006968, cellular defense response	20	1.24E-05
GO:0048522, positive regulation of cellular process	197	2.45E-05
GO:0051704, multi-organism process	90	2.72E-05
GO:0050896, response to stimulus	335	2.63E-05
GO:0042110, T cell activation	29	2.69E-05
GO:0002520, immune system development	47	3.89E-05
GO:0002682, regulation of immune system process	59	3.76E-05
GO:0048534, hemopoietic or lymphoid organ development	45	4.16E-05
GO:0009617, response to bacterium	37	4.34E-05
GO:0030097, hemopoiesis	42	4.72E-05
GO:0030098, lymphocyte differentiation	25	6.20E-05
GO:0006916, anti-apoptosis	38	6.99E-05
GO:0006954, inflammatory response	51	1.05E-04
GO:0030217, T cell differentiation	19	1.03E-04
GO:0006414, translational elongation	24	1.42E-04
GO:0043066, negative regulation of apoptosis	53	2.38E-04
GO:0002819, regulation of adaptive immune response	17	2.39E-04
GO:0043069, negative regulation of programmed cell death	53	3.39E-04
GO:0060548, negative regulation of cell death	53	3.56E-04
GO:0048872, homeostasis of number of cells	23	3.76E-04
GO:0002521, leukocyte differentiation	27	3.82E-04
GO:0009611, response to wounding	70	3.88E-04
GO:0002237, response to molecule of bacterial origin	21	3.85E-04
GO:0048523, negative regulation of cellular process	172	5.00E-04
GO:0048519, negative regulation of biological process	185	4.89E-04
GO:0007243, protein kinase cascade	53	6.53E-04

GO:0002822, regulation of adaptive immune response based on somatic recombination of immune receptors built from immunoglobulin superfamily domains	16	7.02E-04
GO:0032496, response to lipopolysaccharide	19	9.41E-04
GO:0009615, response to virus	23	0.00128
GO:0034097, response to cytokine stimulus	19	0.00131
GO:0002684, positive regulation of immune system process	38	0.00131
GO:0006915, apoptosis	73	0.00350
GO:0010033, response to organic substance	84	0.00362
GO:0016265, death	84	0.00412
GO:0050776, regulation of immune response	35	0.00513
GO:0008219, cell death	83	0.00515
GO:0012501, programmed cell death	73	0.00510
GO:0007259, JAK-STAT cascade	12	0.00631
GO:0006357, regulation of transcription from RNA polymerase II promoter	83	0.00704
GO:0009893, positive regulation of metabolic process	100	0.00879
GO:0043068, positive regulation of programmed cell death	55	0.00890
GO:0002252, immune effector process	24	0.00902
GO:0007242, intracellular signaling cascade	129	0.00966
GO:0010942, positive regulation of cell death	55	0.00949
GO:0031325, positive regulation of cellular metabolic process	96	0.00945
GO:0009605, response to external stimulus	99	0.00940

Supplementary Data

Improvement in human immune function with changes in intestinal microbiota by *Salacia reticulata* extract ingestion

Yuriko Oda, Fumitaka Ueda, Masanori Utsuyama, Asuka Kamei, Chihaya Kakinuma, Keiko Abe, and Katsuiku Hirokawa

S1 Table. GO terms with Benjamini and Hochberg FDR-corrected *p*-value of <0.01

**Term**	**Count**	**Benjamini**
GO:0002376, immune system process	181	4.36E-27
GO:0006955, immune response	135	1.28E-22
GO:0045321, leukocyte activation	54	8.33E-10
GO:0006952, defense response	95	1.23E-08
GO:0001775, cell activation	57	1.54E-08
GO:0051707, response to other organism	56	6.11E-08
GO:0046649, lymphocyte activation	44	8.47E-08
GO:0009607, response to biotic stimulus	64	1.00E-06
GO:0006950, response to stress	189	1.88E-06
GO:0048518, positive regulation of biological process	219	2.30E-06
GO:0043067, regulation of programmed cell death	106	4.87E-06
GO:0042981, regulation of apoptosis	105	5.10E-06
GO:0010941, regulation of cell death	106	4.90E-06
GO:0006968, cellular defense response	20	1.24E-05
GO:0048522, positive regulation of cellular process	197	2.45E-05
GO:0051704, multi-organism process	90	2.72E-05
GO:0050896, response to stimulus	335	2.63E-05
GO:0042110, T cell activation	29	2.69E-05
GO:0002520, immune system development	47	3.89E-05
GO:0002682, regulation of immune system process	59	3.76E-05
GO:0048534, hemopoietic or lymphoid organ development	45	4.16E-05
GO:0009617, response to bacterium	37	4.34E-05
GO:0030097, hemopoiesis	42	4.72E-05
GO:0030098, lymphocyte differentiation	25	6.20E-05
GO:0006916, anti-apoptosis	38	6.99E-05
GO:0006954, inflammatory response	51	1.05E-04
GO:0030217, T cell differentiation	19	1.03E-04
GO:0006414, translational elongation	24	1.42E-04
GO:0043066, negative regulation of apoptosis	53	2.38E-04
GO:0002819, regulation of adaptive immune response	17	2.39E-04
GO:0043069, negative regulation of programmed cell death	53	3.39E-04
GO:0060548, negative regulation of cell death	53	3.56E-04
GO:0048872, homeostasis of number of cells	23	3.76E-04
GO:0002521, leukocyte differentiation	27	3.82E-04
GO:0009611, response to wounding	70	3.88E-04
GO:0002237, response to molecule of bacterial origin	21	3.85E-04
GO:0048523, negative regulation of cellular process	172	5.00E-04
GO:0048519, negative regulation of biological process	185	4.89E-04
GO:0007243, protein kinase cascade	53	6.53E-04

GO:0002822, regulation of adaptive immune response based on somatic recombination of immune receptors built from immunoglobulin superfamily domains	16	7.02E-04
GO:0032496, response to lipopolysaccharide	19	9.41E-04
GO:0009615, response to virus	23	0.00128
GO:0034097, response to cytokine stimulus	19	0.00131
GO:0002684, positive regulation of immune system process	38	0.00131
GO:0006915, apoptosis	73	0.00350
GO:0010033, response to organic substance	84	0.00362
GO:0016265, death	84	0.00412
GO:0050776, regulation of immune response	35	0.00513
GO:0008219, cell death	83	0.00515
GO:0012501, programmed cell death	73	0.00510
GO:0007259, JAK-STAT cascade	12	0.00631
GO:0006357, regulation of transcription from RNA polymerase II promoter	83	0.00704
GO:0009893, positive regulation of metabolic process	100	0.00879
GO:0043068, positive regulation of programmed cell death	55	0.00890
GO:0002252, immune effector process	24	0.00902
GO:0007242, intracellular signaling cascade	129	0.00966
GO:0010942, positive regulation of cell death	55	0.00949
GO:0031325, positive regulation of cellular metabolic process	96	0.00945
GO:0009605, response to external stimulus	99	0.00940
