# Supplementary material for: Improvement in Human Immune Function with Changes in Intestinal Microbiota by Salacia reticulata Extract Ingestion: A Randomized Placebo-Controlled Trial
Source: PLoS One. 2015 Dec 2;10(12):e0142909. doi: 10.1371/journal.pone.0142909 (PMC4667990; doi:10.1371/journal.pone.0142909)
Supplement: S2 Table — (PDF) [file pone.0142909.s005.pdf]

Supplementary Data

Improvement in human immune function with changes in intestinal microbiota by *Salacia reticulata* extract ingestion

Yuriko Oda, Fumitaka Ueda, Masanori Utsuyama, Asuka Kamei, Chihaya Kakinuma, Keiko Abe, and Katsuiku Hirokawa

S2 Table. Canonical pathway

Pathways given by IPS were sorted in order of enrichment in descending order.

Shown are the pathways with both a *p*-value <0.05 and a |*z*-score| >1.5.

**Ingenuity canonical pathway**	**−Log(*p*value)**	***z*Score**
Interferon signaling	6.56E+00	1.897
iCOS-iCOSL signaling in T-helper cells	3.75E+00	–1.897
Role of NFAT in regulation of the immune response	3.73E+00	–1.698
fMLP signaling in neutrophils	3.22E+00	–2.111
Production of nitric oxide and reactive oxygen species in macrophages	3.01E+00	1.606
EIF2 signaling	2.90E+00	1.508
Leukocyte extravasation signaling	2.53E+00	–1.807
CD28 signaling in T-helper cells	2.40E+00	–2.111
Death receptor signaling	2.37E+00	2.111
p70S6K signaling	2.36E+00	–2.496
IL-9 signaling	2.31E+00	–1.633
PTEN signaling	1.98E+00	2.111
Activation of IRF by cytosolic pattern recognition receptors	1.97E+00	2.121
Retinoic acid-mediated apoptosis signaling	1.97E+00	2.121
VEGF signaling	1.92E+00	–1.89

IL-2 signaling	1.91E+00	–1.89
FcγRIIB signaling in B lymphocytes	1.91E+00	–1.633
LXR/RXR activation	1.90E+00	–1.732
Fcγ receptor-mediated phagocytosis in macrophages and monocytes	1.89E+00	–2.53
Renal cell carcinoma signaling	1.72E+00	–1.633
α-Adrenergic signaling	1.65E+00	–1.633
HGF signaling	1.56E+00	–1.89
Glioma signaling	1.44E+00	–2.121
