# Supplementary material for: Improvement in Human Immune Function with Changes in Intestinal Microbiota by Salacia reticulata Extract Ingestion: A Randomized Placebo-Controlled Trial
Source: PLoS One. 2015 Dec 2;10(12):e0142909. doi: 10.1371/journal.pone.0142909 (PMC4667990; doi:10.1371/journal.pone.0142909)
Supplement: S3 Table — (PDF) [file pone.0142909.s006.pdf]

Supplementary Data

Improvement in human immune function with changes in intestinal microbiota by *Salacia reticulata* extract ingestion

Yuriko Oda, Fumitaka Ueda, Masanori Utsuyama, Asuka Kamei, Chihaya Kakinuma, Keiko Abe, and Katsuiku Hirokawa

S3 Table. Up-regulated genes

The obtained data were normalized using the DFW method. Intergroup comparison of the DFW-normalized data was performed using the RP method. Probe sets with an FDR of <0.05 were extracted.

**Gene Symbol**	**Gene Title**	**UniGene ID**
ABHD2	abhydrolase domain containing 2	Hs.122337
ACADM	acyl-CoA dehydrogenase, C-4 to C-12 straight chain	Hs.445040
ACAT1	acetyl-CoA acetyltransferase 1	Hs.232375
ACTR6	ARP6 actin-related protein 6 homolog (yeast)	Hs.115088
ADORA3	adenosine A3 receptor	Hs.281342
AIF1	allograft inflammatory factor 1	Hs.76364
AIM2	absent in melanoma 2	Hs.281898
AIMP1	aminoacyl tRNA synthetase complex-interacting multi-functional protein 1	Hs.591680
AK1	adenylate kinase 1	Hs.175473
ALCAM	activated leukocyte cell adhesion molecule	Hs.591293
ALDH1A1	aldehyde dehydrogenase 1 family, member A1	Hs.76392
ALG13	asparagine-linked glycosylation 13 homolog (*S. cerevisiae*)	Hs.443061
ALPK1	alpha-kinase 1	Hs.652825
ANKRD22	ankyrin repeat domain 22	Hs.217484
ANP32E	acidic (leucine-rich) nuclear phosphoprotein 32 family, member E	Hs.656466
ANXA11	annexin A11	Hs.530291
ANXA3	annexin A3	Hs.480042
AP1S2	adaptor-related protein complex 1, sigma 2 subunit	Hs.653504
APOL6	apolipoprotein L, 6	Hs.257352
AQP9	aquaporin 9	Hs.104624
ARAP1	ArfGAP with RhoGAP domain, ankyrin repeat and PH domain 1	Hs.503165
ARG1	arginase, liver	Hs.440934
ARGLU1	arginine and glutamate rich 1	Hs.724380
ARID1B	AT rich interactive domain 1B (SWI1-like)	Hs.724539
ARL5A	ADP-ribosylation factor-like 5A	Hs.470233
ARPP19	cAMP-regulated phosphoprotein, 19 kDa	Hs.724413
ARRDC4	arrestin domain containing 4	Hs.6093
ASGR2	asialoglycoprotein receptor 2	Hs.654440
ASNSD1	asparagine synthetase domain containing 1	Hs.101364
ASPH	aspartate beta-hydroxylase	Hs.332422
ASPHD2	aspartate beta-hydroxylase domain containing 2	Hs.567547
ATG5	autophagy related 5	Hs.486063
ATP5C1	ATP synthase, H+ transporting, mitochondrial F1 complex, gamma polypeptide 1	Hs.271135
ATP5L	ATP synthase, H+ transporting, mitochondrial Fo complex, subunit G	Hs.486360
ATP5O	ATP synthase, H+ transporting, mitochondrial F1 complex, O subunit	Hs.409140
B3GNT5 /// LOC100505668	UDP-GlcNAc:betaGal beta-1,3-N-acetylglucosaminyltransferase 5 /// uncharacterized LOC100505668	Hs.718506
B4GALT5	UDP-Gal:betaGlcNAc beta 1,4- galactosyltransferase, polypeptide 5	Hs.370487

BATF2	basic leucine zipper transcription factor, ATF-like 2	Hs.124840
BAZ1A	bromodomain adjacent to zinc finger domain, 1A	Hs.509140
BCL2A1	BCL2-related protein A1	Hs.227817
BCL3	B-cell CLL/lymphoma 3	Hs.31210
BCL6	B-cell CLL/lymphoma 6	Hs.478588
BCLAF1	BCL2-associated transcription factor 1	Hs.486542
BID	BH3-interacting domain death agonist	Hs.591054
BIRC2	baculoviral IAP repeat containing 2	Hs.696238
BLOC1S2	biogenesis of lysosomal organelles complex-1, subunit 2	Hs.34906
BNIP3L	BCL2/adenovirus E1B 19 kDa-interacting protein 3-like	Hs.131226
BPI	bactericidal/permeability-increasing protein	Hs.529019
BST1	bone marrow stromal cell antigen 1	Hs.720344
BTK	Bruton agammaglobulinemia tyrosine kinase	Hs.159494
C10orf32	chromosome 10 open reading frame 32	Hs.34492
C11orf75	chromosome 11 open reading frame 75	Hs.438064
C14orf118	chromosome 14 open reading frame 118	Hs.594106
C14orf129	chromosome 14 open reading frame 129	Hs.4104
C14orf2	chromosome 14 open reading frame 2	Hs.109052
C14orf45	chromosome 14 open reading frame 45	Hs.644621
C17orf76-AS1 /// SNORD49A /// SNORD49B /// SNORD65	C17orf76 antisense RNA 1 (non-protein coding) /// small nucleolar RNA, C/D box 49A /// small nucleolar RNA, C/D box 49B /// small nucleolar RNA, C/D box 65	Hs.368934
C18orf32 /// RPL17-C18ORF32	chromosome 18 open reading frame 32 /// RPL17-C18orf32 readthrough	Hs.654638
C19orf59	chromosome 19 open reading frame 59	Hs.709539
C1GALT1C1	C1GALT1-specific chaperone 1	Hs.643920
C1orf9	chromosome 1 open reading frame 9	Hs.204559
C2orf88	chromosome 2 open reading frame 88	Hs.720468
C4orf3	chromosome 4 open reading frame 3	Hs.701808
C4orf32	chromosome 4 open reading frame 32	Hs.23439
C7orf60	chromosome 7 open reading frame 60	Hs.489734
C8orf59	chromosome 8 open reading frame 59	Hs.443072
C9orf72	chromosome 9 open reading frame 72	Hs.493639
CAMP	cathelicidin antimicrobial peptide	Hs.51120
CAPZA1	capping protein (actin filament) muscle Z-line, alpha 1	Hs.514934
CAPZA2	capping protein (actin filament) muscle Z-line, alpha 2	Hs.446123
CARD16	caspase recruitment domain family, member 16	Hs.348365
CASP1	caspase 1, apoptosis-related cysteine peptidase	Hs.2490
CASP3	caspase 3, apoptosis-related cysteine peptidase	Hs.141125
CASP4	caspase 4, apoptosis-related cysteine peptidase	Hs.138378
CASP5	caspase 5, apoptosis-related cysteine peptidase	Hs.213327
CBX3	chromobox homolog 3	Hs.381189
CCDC126	coiled-coil domain containing 126	Hs.232296
CCDC82	coiled-coil domain containing 82	Hs.525088

CCNC	cyclin C	Hs.430646
CCNT2	cyclin T2	Hs.591241
CCR1	chemokine (C-C motif) receptor 1	Hs.301921
CCR2	chemokine (C-C motif) receptor 2	Hs.511794
CCT2	chaperonin containing TCP1, subunit 2 (beta)	Hs.189772
CD177	CD177 molecule	Hs.232165
CD1D	CD1d molecule	Hs.1799
CD274	CD274 molecule	Hs.521989
CD36	CD36 molecule (thrombospondin receptor)	Hs.120949
CD52	CD52 molecule	Hs.276770
CD58	CD58 molecule	Hs.34341
CD86	CD86 molecule	Hs.171182
CDC42EP2	CDC42 effector protein (Rho GTPase binding) 2	Hs.343380
CDK1	cyclin-dependent kinase 1	Hs.334562
CEACAM6	carcinoembryonic antigen-related cell adhesion molecule 6 (non-specific cross reacting antigen)	Hs.466814
CEACAM8	carcinoembryonic antigen-related cell adhesion molecule 8	Hs.41
CEP19	centrosomal protein 19 kDa	Hs.282800
CEP97	centrosomal protein 97 kDa	Hs.444135
CETN3	centrin, EF-hand protein, 3	Hs.591767
CFLAR	CASP8 and FADD-like apoptosis regulator	Hs.390736
CHAC2	ChaC, cation transport regulator homolog 2 (*E. coli*)	Hs.585944
CHKB-CPT1B /// CPT1B	CHKB-CPT1B readthrough (non-protein coding) /// carnitine palmitoyltransferase 1B (muscle)	Hs.439777
CHMP5	charged multivesicular body protein 5	Hs.635313
CHORDC1	cysteine and histidine-rich domain (CHORD) containing 1	Hs.22857
CHPT1	choline phosphotransferase 1	Hs.293077
CHRM3-AS2	CHRM3 antisense RNA 2 (non-protein coding)	Hs.667175
CHURC1	churchill domain containing 1	Hs.325531
CISD2	CDGSH iron sulfur domain 2	Hs.724623
CKAP2 /// IGLC1	Cytoskeleton-associated protein 2 /// Immunoglobulin lambda constant 1 (Mcg marker)	---
CKLF	chemokine-like factor	Hs.15159
CKS2	CDC28 protein kinase regulatory subunit 2	Hs.83758
CLC	Charcot-Leyden crystal protein	Hs.889
CLEC12A	C-type lectin domain family 12, member A	Hs.190519
CLEC2B	C-type lectin domain family 2, member B	Hs.85201
CLEC4A	C-type lectin domain family 4, member A	Hs.504657
CLIC4	chloride intracellular channel 4	Hs.440544
CLNS1A	chloride channel, nucleotide-sensitive, 1A	Hs.430733
CLU	clusterin	Hs.436657
CMC2	COX assembly mitochondrial protein 2 homolog (*S. cerevisiae*)	Hs.388255
CMPK2	cytidine monophosphate (UMP-CMP) kinase 2, mitochondrial	Hs.7155
CNIH4	cornichon homolog 4 (*Drosophila*)	Hs.445890
CNN2	calponin 2	Hs.651512

CNTNAP3 /// CNTNAP3B	contactin-associated protein-like 3 /// contactin-associated protein-like 3B	Hs.658328
COL18A1	collagen, type XVIII, alpha 1	Hs.517356
COMMD10	COMM domain containing 10	Hs.483136
COMMD8	COMM domain containing 8	Hs.23956
COPS2	COP9 constitutive photomorphogenic homolog subunit 2 (*Arabidopsis*)	Hs.369614
COTL1	coactosin-like 1 (*Dictyostelium*)	Hs.660628
COX16	COX16 cytochrome c oxidase assembly homolog (*S. cerevisiae*)	Hs.709581
COX6C	cytochrome c oxidase subunit Vic	Hs.351875
COX7A2	cytochrome c oxidase subunit VIIa polypeptide 2 (liver)	Hs.70312
COX7C	cytochrome c oxidase subunit VIIc	Hs.430075
COX7C	cytochrome c oxidase subunit VIIc	Hs.430075
CPEB4	cytoplasmic polyadenylation element-binding protein 4	Hs.127126
CPNE8	copine VIII	Hs.40910
CRBN	cereblon	Hs.18925
CREB5	CAMP-responsive element-binding protein 5	Hs.437075
CREG1	cellular repressor of E1A-stimulated genes 1	Hs.5710
CSRNP1	cysteine-serine-rich nuclear protein 1	Hs.370950
CSTA	cystatin A (stefin A)	Hs.518198
CUL5	cullin 5	Hs.440320
CXCL10	chemokine (C-X-C motif) ligand 10	Hs.632586
CXCL16	chemokine (C-X-C motif) ligand 16	Hs.724659
CXCR1	chemokine (C-X-C motif) receptor 1	Hs.194778
CXCR2	chemokine (C-X-C motif) receptor 2	Hs.724268
CYBB	cytochrome b-245, beta polypeptide	Hs.292356
CYP1B1	cytochrome P450, family 1, subfamily B, polypeptide 1	Hs.154654
CYSTM1	cysteine-rich transmembrane module containing 1	Hs.529798
DAPP1	dual adaptor of phosphotyrosine and 3-phosphoinositides	Hs.436271
DBI	diazepam-binding inhibitor (GABA receptor modulator, acyl-CoA-binding protein)	Hs.78888
DDX58	DEAD (Asp-Glu-Ala-Asp) box polypeptide 58	Hs.190622
DDX60L	DEAD (Asp-Glu-Ala-Asp) box polypeptide 60-like	Hs.535011
DEK	DEK oncogene	Hs.484813
DENND4B	DENN/MADD domain containing 4B	Hs.632480
DHRS13	dehydrogenase/reductase (SDR family) member 13	Hs.631760
DNAJA1	DnaJ (Hsp40) homolog, subfamily A, member 1	Hs.445203
DNAJA4	DnaJ (Hsp40) homolog, subfamily A, member 4	Hs.513053
DNAJB9	DnaJ (Hsp40) homolog, subfamily B, member 9	Hs.6790
DNASE1L1	deoxyribonuclease I-like 1	Hs.401929
DOCK4	dedicator of cytokinesis 4	Hs.654652
DPH3	DPH3, KTI11 homolog (*S. cerevisiae*)	Hs.388087
DPM1	dolichyl-phosphate mannosyltransferase polypeptide 1, catalytic subunit	Hs.654951
DPY30	dpy-30 homolog (*C. elegans*)	Hs.531788
DR1	down-regulator of transcription 1, TBP-binding (negative cofactor 2)	Hs.348418
DRAM1	DNA-damage regulated autophagy modulator 1	Hs.525634
DSC2	desmocollin 2	Hs.95612

DSE	dermatan sulfate epimerase	Hs.458358
DUSP1	dual specificity phosphatase 1	Hs.171695
DYNLT1	dynein, light chain, Tctex-type 1	Hs.445999
ECT2	epithelial cell transforming sequence 2 oncogene	Hs.518299
EEF1E1	eukaryotic translation elongation factor 1 epsilon 1	Hs.602353
EGLN1	egl nine homolog 1 (*C. elegans*)	Hs.444450
EIF1AY	eukaryotic translation initiation factor 1A, Y-linked	Hs.461178
EIF2S1	eukaryotic translation initiation factor 2, subunit 1 alpha, 35 kDa	Hs.151777
EIF3E	eukaryotic translation initiation factor 3, subunit E	Hs.405590
EIF4E	eukaryotic translation initiation factor 4E	Hs.249718
EIF4E3	eukaryotic translation initiation factor 4E family member 3	Hs.581355
ELANE	elastase, neutrophil expressed	Hs.99863
ELL2	elongation factor, RNA polymerase II, 2	Hs.192221
EMR1	egf-like module containing, mucin-like, hormone receptor-like 1	Hs.2375
ENTPD1	ectonucleoside triphosphate diphosphohydrolase 1	Hs.576612
EPSTI1	epithelial stromal interaction 1 (breast)	Hs.546467
ERAP1	endoplasmic reticulum aminopeptidase 1	Hs.716426
ERH	enhancer of rudimentary homolog (*Drosophila*)	Hs.509791
ERV3-2	endogenous retrovirus group 3, member 2	---
ETV7	ets variant 7	Hs.272398
EXOC6	exocyst complex component 6	Hs.655657
F2RL1	coagulation factor II (thrombin) receptor-like 1	Hs.154299
FAM102B	family with sequence similarity 102, member B	Hs.200230
FAM126B	family with sequence similarity 126, member B	Hs.24701
FAM160B1	family with sequence similarity 160, member B1	Hs.192619
FAM198B	family with sequence similarity 198, member B	Hs.567498
FAM212B	family with sequence similarity 212, member B	Hs.193406
FAM26F	family with sequence similarity 26, member F	Hs.381220
FAM35A	family with sequence similarity 35, member A	Hs.500419
FAM96A	family with sequence similarity 96, member A	Hs.439548
FAR1	fatty acyl CoA reductase 1	Hs.501991
FAR2	fatty acyl CoA reductase 2	Hs.724583
FAS	Fas (TNF receptor superfamily, member 6)	Hs.244139
FBXO6	F-box protein 6	Hs.464419
FCAR	Fc fragment of IgA, receptor for	Hs.659872
FCER1A	Fc fragment of IgE, high affinity I, receptor for; alpha polypeptide	Hs.897
FCGR1A /// FCGR1B /// FCGR1C	Fc fragment of IgG, high affinity Ia, receptor (CD64) /// Fc fragment of IgG, high affinity Ib, receptor (CD64) /// Fc fragment of IgG, high affinity Ic, receptor (CD64), pseudogene	Hs.77424
FCGR1B	Fc fragment of IgG, high affinity Ib, receptor (CD64)	Hs.534956
FCHO2	FCH domain only 2	Hs.724589
FFAR2	free fatty acid receptor 2	Hs.248056
FGF13	fibroblast growth factor 13	Hs.6540
FKBP1B	FK506-binding protein 1B, 12.6 kDa	Hs.709461
FLJ39051	uncharacterized LOC399972	Hs.585206
FLJ45340	uncharacterized LOC402483	Hs.465593

FLJ45340 /// FLJ45445 /// LOC100128326 /// LOC100132050 /// LOC100287894 /// LOC100289306 /// LOC100506479 /// LOC100508632 /// LOC100652945 /// LOC100653241 /// LOC100653346 /// LOC729737	uncharacterized LOC402483 /// uncharacterized LOC399844 /// putative uncharacterized protein FLJ44672-like /// uncharacterized LOC100132050 /// uncharacterized LOC100287894 /// uncharacterized LOC100289306 /// uncharacterized LOC100506479 /// uncharacterized LOC100508632 /// uncharacterized LOC100652945 /// uncharacterized LOC100653241 /// uncharacterized LOC100653346 /// uncharacterized LOC729737	Hs.465593
FLOT1	flotillin 1	Hs.179986
FLVCR1	feline leukemia virus subgroup C cellular receptor 1	Hs.7055
FMNL2	formin-like 2	Hs.654630
FOLR3	folate receptor 3 (gamma)	Hs.352
FOS	FBJ murine osteosarcoma viral oncogene homolog	Hs.25647
FPR2	formyl peptide receptor 2	Hs.99855
FRAT2	frequently rearranged in advanced T-cell lymphomas 2	Hs.140720
FRMD3	FERM domain containing 3	Hs.709357
FUNDC2	FUN14 domain containing 2	Hs.356050
GAS5 /// SNORD44 /// SNORD47 /// SNORD76 /// SNORD77 /// SNORD79 /// SNORD80 /// SNORD81	growth arrest-specific 5 (non-protein coding) /// small nucleolar RNA, C/D box 44 /// small nucleolar RNA, C/D box 47 /// small nucleolar RNA, C/D box 76 /// small nucleolar RNA, C/D box 77 /// small nucleolar RNA, C/D box 79 /// small nucleolar RNA, C/D box 80 /// small nucleolar RNA, C/D box 81	Hs.531856
GBP2	guanylate-binding protein 2, interferon-inducible	---
GBP3	guanylate-binding protein 3	Hs.720167
GCH1	GTP cyclohydrolase 1	Hs.86724
GIMAP2	GTPase, IMAP family member 2	Hs.647071
GK	glycerol kinase	Hs.1466
GK3P	glycerol kinase 3 pseudogene	Hs.654557
GLRX	glutaredoxin (thioltransferase)	Hs.28988
GLT1D1	glycosyltransferase 1 domain containing 1	Hs.655668
GM2A	GM2 ganglioside activator	Hs.483873
GM2A	GM2 ganglioside activator	Hs.483873
GMFB	glia maturation factor, beta	Hs.151413

GMNN	geminin, DNA replication inhibitor	Hs.234896
GNB4	guanine nucleotide-binding protein (G protein), beta polypeptide 4	Hs.173030
GNG10	guanine nucleotide-binding protein (G protein), gamma 10	Hs.534196
GNG11	guanine nucleotide-binding protein (G protein), gamma 11	Hs.83381
GNL3 /// SNORD19B	guanine nucleotide-binding protein-like 3 (nucleolar) /// small nucleolar RNA, C/D box 19B	Hs.313544
GPR160	G protein-coupled receptor 160	Hs.231320
GPR65	G protein-coupled receptor 65	Hs.513440
GPR84	G protein-coupled receptor 84	Hs.306199
GSN	gelsolin	Hs.522373
GSPT1	G1 to S phase transition 1	Hs.528780
GUK1	guanylate kinase 1	Hs.376933
HAL	histidine ammonia-lyase	Hs.190783
HAT1	histone acetyltransferase 1	Hs.632532
HAUS1	HAUS augmin-like complex, subunit 1	Hs.436617
HAUS4 /// MIR4707	HAUS augmin-like complex, subunit 4 /// microRNA 4707	Hs.442782
HCG27	HLA complex group 27 (non-protein coding)	Hs.659818
HEBP1	heme-binding protein 1	Hs.642618
HEMGN	Hemogen	Hs.176626
HERC5	HECT and RLD domain containing E3 ubiquitin protein ligase 5	Hs.26663
HIGD1A	HIG1 hypoxia inducible domain family, member 1A	Hs.593134
HINT1	histidine triad nucleotide-binding protein 1	Hs.483305
HIPK3	homeodomain-interacting protein kinase 3	Hs.201918
HIST1H2BC /// HIST1H2BE /// HIST1H2BF /// HIST1H2BG /// HIST1H2BI	histone cluster 1, H2bc /// histone cluster 1, H2be /// histone cluster 1, H2bf /// histone cluster 1, H2bg /// histone cluster 1, H2bi	Hs.658713
HIST2H2BE	histone cluster 2, H2be	Hs.2178
HLA-DPA1	major histocompatibility complex, class II, DP alpha 1	Hs.347270
HLX	H2.0-like homeobox	Hs.74870
HMGB1	high mobility group box 1	Hs.434102
HMGB2	high mobility group box 2	Hs.434953
HNMT	histamine N-methyltransferase	Hs.42151
HPGD	hydroxyprostaglandin dehydrogenase 15-(NAD)	Hs.596913
HPSE	heparanase	Hs.44227
HSBP1	heat shock factor-binding protein 1	Hs.250899
HSP90AA1	heat shock protein 90 kDa alpha (cytosolic), class A member 1	Hs.525600
HSPA1A /// HSPA1B	heat shock 70 kDa protein 1A /// heat shock 70 kDa protein 1B	Hs.274402
HSPA6	heat shock 70 kDa protein 6 (HSP70B')	Hs.654614
HSPE1-MOB4 /// MOB4	HSPE1-MOB4 readthrough /// MOB family member 4, phocein	Hs.645458
HSPH1	heat shock 105 kDa/110 kDa protein 1	Hs.36927
IDO1	indoleamine 2,3-dioxygenase 1	Hs.840
IFI35	interferon-induced protein 35	Hs.632258

IFI44	interferon-induced protein 44	Hs.82316
IFI6	interferon, alpha-inducible protein 6	Hs.523847
IFIH1	interferon induced with helicase C domain 1	Hs.163173
IFIT1	interferon-induced protein with tetratricopeptide repeats 1	Hs.20315
IFIT2	interferon-induced protein with tetratricopeptide repeats 2	Hs.437609
IFIT3	interferon-induced protein with tetratricopeptide repeats 3	Hs.47338
IFIT5	interferon-induced protein with tetratricopeptide repeats 5	Hs.252839
IGF2BP3	insulin-like growth factor 2 mRNA-binding protein 3	Hs.700696
IGJ	immunoglobulin J polypeptide, linker protein for immunoglobulin alpha and mu polypeptides	Hs.643431
IGK@ /// IGKC	immunoglobulin kappa locus /// immunoglobulin kappa constant	Hs.721572
IGKC	Immunoglobulin kappa constant	Hs.449621
IGKC /// LOC642838	immunoglobulin kappa constant /// ig kappa chain V-I region Walker-like	Hs.574701
IL13RA1	interleukin 13 receptor, alpha 1	Hs.496646
IL15	interleukin 15	Hs.654378
IL1B	interleukin 1, beta	Hs.126256
IL1R2	interleukin 1 receptor, type II	Hs.25333
IL1RN	interleukin 1 receptor antagonist	Hs.81134
IMPA1	inositol(myo)-1(or 4)-monophosphatase 1	Hs.656694
IPO11 /// LRRC70	importin 11 /// leucine rich repeat containing 70	Hs.482269
IRF1	interferon regulatory factor 1	Hs.436061
ISCA1	iron-sulfur cluster assembly 1 homolog (*S. cerevisiae*)	Hs.449291
ISG15	ISG15 ubiquitin-like modifier	Hs.458485
JAK2	Janus kinase 2	Hs.656213
JUNB	jun B proto-oncogene	Hs.25292
KAZN	kazrin, periplakin-interacting protein	Hs.368823
KBTBD7	kelch repeat and BTB (POZ) domain containing 7	Hs.63841
KCNJ15	potassium inwardly-rectifying channel, subfamily J, member 15	Hs.411299
KCNJ2	potassium inwardly-rectifying channel, subfamily J, member 2	Hs.1547
KIAA0101	KIAA0101	Hs.81892
KIAA0391 /// PSMA6	KIAA0391 /// proteasome (prosome, macropain) subunit, alpha type, 6	Hs.446260
KIAA1033	KIAA1033	Hs.12144
KIAA1598	KIAA1598	Hs.501140
KLC3	kinesin light chain 3	Hs.298079
KLHL2	kelch-like 2, Mayven (*Drosophila*)	Hs.388668
KRCC1	lysine-rich coiled-coil 1	Hs.469254
KREMEN1	kringle-containing transmembrane protein 1	Hs.229335
KTN1	kinectin 1 (kinesin receptor)	Hs.509414
KYNU	kynureninase	Hs.470126
LACTB	lactamase, beta	Hs.410388
LAIR2	leukocyte-associated immunoglobulin-like receptor 2	Hs.43803
LAMTOR3	late endosomal/lysosomal adaptor, MAPK and MTOR activator 3	Hs.723128
LAP3	leucine aminopeptidase 3	Hs.570791
LCN2	lipocalin 2	Hs.204238

LGALS2	lectin, galactoside-binding, soluble, 2	Hs.531776
LGALSL	lectin, galactoside-binding-like	Hs.372208
LIMK2	LIM domain kinase 2	Hs.474596
LIN7A	lin-7 homolog A (*C. elegans*)	Hs.624171
LINC00189	long intergenic non-protein-coding RNA 189	Hs.720711
LOC100129518 /// SOD2	uncharacterized LOC100129518 /// superoxide dismutase 2, mitochondrial	Hs.487046
LOC100505812	uncharacterized LOC100505812	Hs.614136
LOC100505991 /// PIGC	uncharacterized LOC100505991 /// phosphatidylinositol glycan anchor biosynthesis, class C	Hs.188456
LOC100506903 /// LOC100652783 /// LOC100652799 /// LOC100653060 /// LOC728093	putative POM121-like protein 1-like /// putative POM121-like protein 1-like /// putative POM121-like protein 1-like /// putative POM121-like protein 1-like /// putative POM121-like protein 1-like	---
LOC202025	uncharacterized LOC202025	---
LOC284454	uncharacterized LOC284454	Hs.436426
LOC389834 /// MAFIP /// TEKT4P2	ankyrin repeat domain 57 pseudogene /// MAFF-interacting protein (pseudogene) /// tektin 4 pseudogene 2	Hs.720653
LOC642838	Ig kappa chain V-I region Walker-like	Hs.721572
LOC643072	uncharacterized LOC643072	Hs.632541
LPAR6	lysophosphatidic acid receptor 6	Hs.123464
LPCAT2	lysophosphatidylcholine acyltransferase 2	Hs.460857
LRRK2	leucine-rich repeat kinase 2	Hs.187636
LSM1	LSM1 homolog, U6 small nuclear RNA associated (*S. cerevisiae*)	Hs.425311
LSM5	LSM5 homolog, U6 small nuclear RNA associated (*S. cerevisiae*)	Hs.424908
LY96	lymphocyte antigen 96	Hs.660766
LYSMD2	LysM, putative peptidoglycan-binding, domain containing 2	Hs.603629
LYZ	lysozyme	Hs.524579
MAFB	v-maf musculoaponeurotic fibrosarcoma oncogene homolog B (avian)	Hs.169487
MAFF	v-maf musculoaponeurotic fibrosarcoma oncogene homolog F (avian)	Hs.517617
MAK	male germ cell-associated kinase	Hs.446125
MANEA	mannosidase, endo-alpha	Hs.533323
MANSC1	MANSC domain containing 1	Hs.591145
MAPK14	mitogen-activated protein kinase 14	Hs.485233
MARCKS	myristoylated alanine-rich protein kinase C substrate	Hs.519909
MCL1	myeloid cell leukemia sequence 1 (BCL2-related)	Hs.632486
MCTP1	multiple C2 domains, transmembrane 1	Hs.655087
MCTS1	malignant T cell amplified sequence 1	Hs.102696
MED21	mediator complex subunit 21	Hs.286145
MGST1	microsomal glutathione S-transferase 1	Hs.389700
MKRN1	makorin ring finger protein 1	Hs.490347
MMP8	matrix metallopeptidase 8 (neutrophil collagenase)	Hs.161839

MRFAP1L1	Morf4 family-associated protein 1-like 1	Hs.593159
MRPL15	mitochondrial ribosomal protein L15	Hs.18349
MRPL3	mitochondrial ribosomal protein L3	Hs.205163
MRPL32	mitochondrial ribosomal protein L32	Hs.50252
MRPL42	mitochondrial ribosomal protein L42	Hs.199579
MRPL47	mitochondrial ribosomal protein L47	Hs.283734
MRPS28	mitochondrial ribosomal protein S28	Hs.521124
MRPS33	mitochondrial ribosomal protein S33	Hs.416207
MS4A3	membrane-spanning 4-domains, subfamily A, member 3 (hematopoietic cell-specific)	Hs.99960
MS4A4A	membrane-spanning 4-domains, subfamily A, member 4A	Hs.325960
MS4A6A	membrane-spanning 4-domains, subfamily A, member 6A	Hs.523702
MS4A7	membrane-spanning 4-domains, subfamily A, member 7	Hs.530735
MSRB1	methionine sulfoxide reductase B1	Hs.655346
MTHFD2	methylenetetrahydrofolate dehydrogenase (NADP+ dependent) 2, methenyltetrahydrofolate cyclohydrolase	Hs.469030
MTMR11	myotubularin-related protein 11	Hs.425144
MUTED-TXNDC5 /// TXNDC5	MUTED-TXNDC5 readthrough (non-protein coding) /// thioredoxin domain containing 5 (endoplasmic reticulum)	Hs.150837
MX1	myxovirus (influenza virus) resistance 1, interferon-inducible protein p78 (mouse)	Hs.517307
MXD1	MAX dimerization protein 1	Hs.468908
MYL4	myosin, light chain 4, alkali; atrial, embryonic	Hs.463300
MYOF	myoferlin	Hs.602086
MZB1	marginal zone B and B1 cell-specific protein	Hs.409563
NAB1	NGFI-A-binding protein 1 (EGR1-binding protein 1)	Hs.723892
NAMPT	nicotinamide phosphoribosyltransferase	Hs.489615
NAT1	N-acetyltransferase 1 (arylamine N-acetyltransferase)	Hs.591847
NBN	nibrin	Hs.492208
NCF4	neutrophil cytosolic factor 4, 40 kDa	Hs.474781
NDUFA4	NADH dehydrogenase (ubiquinone) 1 alpha subcomplex, 4, 9 kDa	Hs.50098
NDUFA5	NADH dehydrogenase (ubiquinone) 1 alpha subcomplex, 5, 13 kDa	Hs.651219
NDUFA6	NADH dehydrogenase (ubiquinone) 1 alpha subcomplex, 6, 14 kDa	Hs.274416
NDUFB1	NADH dehydrogenase (ubiquinone) 1 beta subcomplex, 1, 7 kDa	Hs.183435
NDUFB2	NADH dehydrogenase (ubiquinone) 1 beta subcomplex, 2, 8 kDa	Hs.655788
NDUFB3	NADH dehydrogenase (ubiquinone) 1 beta subcomplex, 3, 12 kDa	Hs.109760
NFKBIZ	nuclear factor of kappa light polypeptide gene enhancer in B-cells inhibitor, zeta	Hs.319171
NFXL1	nuclear transcription factor, X-box-binding-like 1	Hs.646325
NIPSNAP3A	nipsnap homolog 3A (*C. elegans*)	Hs.530275
NMD3	NMD3 homolog (*S. cerevisiae*)	Hs.598836
NMI	N-myc (and STAT) interactor	Hs.54483
NOD2	nucleotide-binding oligomerization domain containing 2	Hs.592072
NRBF2	nuclear receptor-binding factor 2	Hs.449628
NRG1	neuregulin 1	Hs.453951
NUDT4	nudix (nucleoside diphosphate linked moiety X)-type motif 4	Hs.506325
NXT2	nuclear transport factor 2-like export factor 2	Hs.25010

OAS1	2′-5′-oligoadenylate synthetase 1, 40/46 kDa	Hs.524760
OAS3	2′-5′-oligoadenylate synthetase 3, 100 kDa	Hs.528634
OASL	2′-5′-oligoadenylate synthetase-like	Hs.118633
OPTN	optineurin	Hs.332706
OR52K3P	olfactory receptor, family 52, subfamily K, member 3 pseudogene	Hs.162035
ORC4	origin recognition complex, subunit 4	Hs.558364
OSGIN2	oxidative stress induced growth inhibitor family member 2	Hs.436445
OSTC	oligosaccharyltransferase complex subunit	Hs.445803
P2RY13	purinergic receptor P2Y, G-protein coupled, 13	Hs.546396
P2RY14	purinergic receptor P2Y, G-protein coupled, 14	Hs.2465
PADI2	peptidyl arginine deiminase, type II	Hs.33455
PARP14	poly (ADP-ribose) polymerase family, member 14	Hs.518203
PARP9	poly (ADP-ribose) polymerase family, member 9	Hs.518200
PCGF5	polycomb group ring finger 5	Hs.500512
PCMTD1	protein-L-isoaspartate (D-aspartate) O-methyltransferase domain containing 1	Hs.671268
PCNP	PEST proteolytic signal-containing nuclear protein	Hs.275865
PCTP	phosphatidylcholine transfer protein	Hs.285218
PDCD10	programmed cell death 10	Hs.478150
PDE4B	phosphodiesterase 4B, cAMP-specific	Hs.198072
PET100	PET100 homolog (*S. cerevisiae*)	---
PF4V1	platelet factor 4 variant 1	Hs.72933
PFDN5	prefoldin subunit 5	Hs.655327
PFKFB3	6-phosphofructo-2-kinase/fructose-2,6-biphosphatase 3	Hs.195471
PGK1	Phosphoglycerate kinase 1	Hs.78771
PHC2	polyhomeotic homolog 2 (*Drosophila*)	Hs.524271
PHTF2	putative homeodomain transcription factor 2	Hs.203965
PKN2	protein kinase N2	Hs.440833
PLBD1	phospholipase B domain containing 1	Hs.131933
PLEKHA3	pleckstrin homology domain containing, family A (phosphoinositide-binding specific) member 3	Hs.41086
PLSCR1	phospholipid scramblase 1	Hs.130759
PMAIP1	phorbol-12-myristate-13-acetate-induced protein 1	Hs.96
PNO1	partner of NOB1 homolog (*S. cerevisiae*)	Hs.262858
PNPLA8	patatin-like phospholipase domain containing 8	Hs.617340
POLB	polymerase (DNA directed), beta	Hs.654484
POLR2K	polymerase (RNA) II (DNA directed) polypeptide K, 7.0 kDa	Hs.351475
PPA1	pyrophosphatase (inorganic) 1	Hs.437403
PPBP	pro-platelet basic protein (chemokine (C-X-C motif) ligand 7)	Hs.2164
PPIL4	peptidylprolyl isomerase (cyclophilin)-like 4	Hs.593411
PPP1CB	Protein phosphatase 1, catalytic subunit, beta isozyme	Hs.702907
PPP1R15A	protein phosphatase 1, regulatory subunit 15A	Hs.631593
PPP1R3B	protein phosphatase 1, regulatory subunit 3B	Hs.458513
PPP4R2	protein phosphatase 4, regulatory subunit 2	Hs.431092
PRDX4	peroxiredoxin 4	Hs.83383
PRKAR2B	protein kinase, cAMP-dependent, regulatory, type II, beta	Hs.433068
PRPF39	PRP39 pre-mRNA processing factor 39 homolog (*S. cerevisiae*)	Hs.274337

PRRG4	proline rich Gla (G-carboxyglutamic acid) 4 (transmembrane)	Hs.471695
PSMA2	proteasome (prosome, macropain) subunit, alpha type, 2	Hs.333786
PSMA3	proteasome (prosome, macropain) subunit, alpha type, 3	Hs.558799
PSMA4	proteasome (prosome, macropain) subunit, alpha type, 4	Hs.251531
PSMC6	proteasome (prosome, macropain) 26S subunit, ATPase, 6	Hs.156171
PSME2	proteasome (prosome, macropain) activator subunit 2 (PA28 beta)	Hs.434081
PSPH	phosphoserine phosphatase	Hs.512656
PSTPIP2	proline-serine-threonine phosphatase-interacting protein 2	Hs.567384
PTAR1	protein prenyltransferase alpha subunit repeat containing 1	Hs.494100
PTEN	phosphatase and tensin homolog	Hs.500466
PTGS2	prostaglandin-endoperoxide synthase 2 (prostaglandin G/H synthase and cyclooxygenase)	Hs.196384
PTPRN2	protein tyrosine phosphatase, receptor type, N polypeptide 2	Hs.490789
PYGL	phosphorylase, glycogen, liver	Hs.282417
QKI	QKI, KH domain containing, RNA binding	Hs.510324
RAB18	RAB18, member RAS oncogene family	Hs.406799
RAB20	RAB20, member RAS oncogene family	Hs.724759
RAB33B	RAB33B, member RAS oncogene family	Hs.591679
RALB	v-ral simian leukemia viral oncogene homolog B (ras related; GTP-binding protein)	Hs.469820
RAP1GAP	RAP1 GTPase-activating protein	Hs.148178
RAP2A	RAP2A, member of RAS oncogene family	Hs.508480
RAP2C	RAP2C, member of RAS oncogene family	Hs.119889
RB1CC1	RB1-inducible coiled-coil 1	Hs.196102
RBBP8	Retinoblastoma-binding protein 8	Hs.546282
RBP7	retinol-binding protein 7, cellular	Hs.422688
RBX1	ring-box 1, E3 ubiquitin protein ligase	Hs.474949
RCHY1	ring finger and CHY zinc finger domain containing 1, E3 ubiquitin protein ligase	Hs.48297
RETN	resistin	Hs.283091
RFC5	replication factor C (activator 1) 5, 36.5 kDa	Hs.506985
RFX2	regulatory factor X, 2 (influences HLA class II expression)	Hs.465709
RGS10	regulator of G-protein signaling 10	Hs.501200
RGS18	regulator of G-protein signaling 18	Hs.440890
RHOQ	ras homolog family member Q	Hs.709193
RHOT1	ras homolog family member T1	Hs.655325
RHOU	ras homolog family member U	Hs.647774
RIT1	Ras-like without CAAX 1	Hs.491234
RNASE2	ribonuclease, RNase A family, 2 (liver, eosinophil-derived neurotoxin)	Hs.728
RNASE6	ribonuclease, RNase A family, k6	Hs.23262
RNF11	ring finger protein 11	Hs.309641
RNF144B	ring finger protein 144B	Hs.148741
RNF24	ring finger protein 24	Hs.589884
RP2	retinitis pigmentosa 2 (X-linked recessive)	Hs.44766
RPAP3	RNA polymerase II-associated protein 3	Hs.437855
RPIA	ribose 5-phosphate isomerase A	Hs.469264
RPL21 ///	ribosomal protein L21 /// ribosomal protein L21 pseudogene 28 /// small nucleolar	Hs.381123

RPL21P28 /// SNORA27 /// SNORD102	RNA, H/ACA box 27 /// small nucleolar RNA, C/D box 102	
RPL22L1	ribosomal protein L22-like 1	Hs.380933
RPL24	ribosomal protein L24	Hs.649475
RPL26L1	ribosomal protein L26-like 1	Hs.546390
RPL27	ribosomal protein L27	Hs.514196
RPL31	ribosomal protein L31	Hs.469473
RPL35	ribosomal protein L35	Hs.182825
RPL35A	ribosomal protein L35a	Hs.529631
RPL41	ribosomal protein L41	Hs.157160
RPS15A	ribosomal protein S15a	Hs.370504
RPS27L	ribosomal protein S27-like	Hs.108957
RRM2	ribonucleotide reductase M2	Hs.226390
RSAD2	radical S-adenosyl methionine domain containing 2	Hs.17518
RSL24D1	ribosomal L24 domain containing 1	Hs.274772
RTP4	receptor (chemosensory) transporter protein 4	Hs.43388
RWDD1	RWD domain containing 1	Hs.532164
S100A12	S100 calcium-binding protein A12	Hs.19413
S100A8	S100 calcium-binding protein A8	Hs.416073
SAMD8	sterile alpha motif domain containing 8	Hs.663616
SAMD9L	sterile alpha motif domain containing 9-like	Hs.489118
SAMSN1	SAM domain, SH3 domain and nuclear localization signals 1	Hs.473341
SAP30	Sin3A-associated protein, 30 kDa	Hs.591715
SAR1B	SAR1 homolog B (*S. cerevisiae*)	Hs.432984
SBF2	SET-binding factor 2	Hs.577252
SCARF1	scavenger receptor class F, member 1	Hs.647430
SCO2	SCO cytochrome oxidase deficient homolog 2 (yeast)	Hs.180903
SCOC	short coiled-coil protein	Hs.480815
SDPR	serum deprivation response	Hs.26530
SEC11C	SEC11 homolog C (*S. cerevisiae*)	Hs.45107
SEC62	SEC62 homolog (*S. cerevisiae*)	Hs.592561
SECTM1	secreted and transmembrane 1	Hs.558009
SELT	selenoprotein T	Hs.369052
SERP1	stress-associated endoplasmic reticulum protein 1	Hs.518326
SERPINB1	serpin peptidase inhibitor, clade B (ovalbumin), member 1	Hs.381167
SERPING1	serpin peptidase inhibitor, clade G (C1 inhibitor), member 1	Hs.384598
SESN3	sestrin 3	Hs.659934
SF3B14	splicing factor 3B, 14 kDa subunit	Hs.177861
SGMS2	sphingomyelin synthase 2	Hs.595423
SIAH2	siah E3 ubiquitin protein ligase 2	Hs.477959
SIGLEC5	sialic acid-binding Ig-like lectin 5	Hs.310333
SIPA1L2	signal-induced proliferation-associated 1 like 2	Hs.724620
SIRPB2	signal-regulatory protein beta 2	Hs.721685
SKIL	SKI-like oncogene	Hs.536655
SLC22A16	solute carrier family 22 (organic cation/carnitine transporter), member 16	Hs.520319

SLC22A4	solute carrier family 22 (organic cation/ergothioneine transporter), member 4	Hs.310591
SLC30A1	solute carrier family 30 (zinc transporter), member 1	Hs.519469
SLC31A2	solute carrier family 31 (copper transporters), member 2	Hs.24030
SLC37A4	solute carrier family 37 (glucose-6-phosphate transporter), member 4	Hs.719203
SLC7A7	solute carrier family 7 (amino acid transporter light chain, y + L system), member 7	Hs.513147
SLIRP	SRA stem-loop-interacting RNA-binding protein	Hs.655105
SLK	STE20-like kinase	Hs.591922
SLPI	secretory leukocyte peptidase inhibitor	Hs.517070
SMC4	structural maintenance of chromosomes 4	Hs.58992
SMPDL3A	sphingomyelin phosphodiesterase, acid-like 3A	Hs.486357
SNCA	synuclein, alpha (non A4 component of amyloid precursor)	Hs.21374
SNRPD2	small nuclear ribonucleoprotein D2 polypeptide 16.5 kDa	Hs.515472
SNRPE	small nuclear ribonucleoprotein polypeptide E	Hs.334612
SNRPG	small nuclear ribonucleoprotein polypeptide G	Hs.465167
SNX10	sorting nexin 10	Hs.520714
SOCS1	suppressor of cytokine signaling 1	Hs.50640
SOCS3	suppressor of cytokine signaling 3	Hs.527973
SORT1	sortilin 1	Hs.485195
SPCS3	signal peptidase complex subunit 3 homolog (*S. cerevisiae*)	Hs.42194
SPOPL	speckle-type POZ protein-like	Hs.333297
SREK1	splicing regulatory glutamine/lysine-rich protein 1	Hs.519347
SRSF6	serine/arginine-rich splicing factor 6	Hs.723837
ST20	suppressor of tumorigenicity 20	---
STARD3NL	STARD3 N-terminal like	Hs.309753
STAT1	signal transducer and activator of transcription 1, 91 kDa	Hs.724418
STAT2	signal transducer and activator of transcription 2, 113 kDa	Hs.530595
STK17B	serine/threonine kinase 17b	Hs.88297
STX11	syntaxin 11	Hs.118958
STX3	syntaxin 3	Hs.180711
STX7	syntaxin 7	Hs.593148
SUB1	SUB1 homolog (*S. cerevisiae*)	Hs.229641
SUPT4H1	suppressor of Ty 4 homolog 1 (*S. cerevisiae*)	Hs.439481
TAL1	T-cell acute lymphocytic leukemia 1	Hs.705618
TANK	TRAF family member-associated NFKB activator	Hs.132257
TAP2	transporter 2, ATP-binding cassette, sub-family B (MDR/TAP)	Hs.502
TAX1BP1	Tax1 (human T-cell leukemia virus type I)-binding protein 1	Hs.34576
TBK1	TANK-binding kinase 1	Hs.505874
TCF7L2	transcription factor 7-like 2 (T-cell specific, HMG-box)	Hs.593995
TCN1	transcobalamin I (vitamin B12-binding protein, R binder family)	Hs.2012
TECPR2	tectonin beta-propeller repeat containing 2	Hs.195667
TFAM	transcription factor A, mitochondrial	Hs.642966
TFEC	transcription factor EC	Hs.125962
TGFA	transforming growth factor, alpha	Hs.170009
THBD	thrombomodulin	Hs.2030
TICAM2 ///	Toll-like receptor adaptor molecule 2 /// TMED7-TICAM2 readthrough	Hs.642817

TMED7-TICAM2		
TIFA	TRAF-interacting protein with forkhead-associated domain	Hs.310640
TIMM8B	translocase of inner mitochondrial membrane 8 homolog B (yeast)	Hs.279915
TKT	transketolase	Hs.89643
TLE3	transducin-like enhancer of split 3 (E(sp1) homolog, *Drosophila*)	Hs.287362
TLR1	Toll-like receptor 1	Hs.654532
TLR5	Toll-like receptor 5	Hs.604542
TMA7	translational machinery-associated 7 homolog (*S. cerevisiae*)	Hs.356440
TMCO1	transmembrane and coiled-coil domains 1	Hs.715707
TMEM126A	transmembrane protein 126A	Hs.533725
TMEM126B	transmembrane protein 126B	Hs.525063
TMEM140	transmembrane protein 140	Hs.724675
TMEM158	transmembrane protein 158 (gene/pseudogene)	Hs.35861
TMEM167A	transmembrane protein 167A	Hs.355606
TMEM170B	transmembrane protein 170B	Hs.146317
TMEM176A	transmembrane protein 176A	Hs.647116
TMTC1	transmembrane and tetratricopeptide repeat containing 1	Hs.401954
TMX1	thioredoxin-related transmembrane protein 1	Hs.125221
TMX3	thioredoxin-related transmembrane protein 3	Hs.440534
TNFAIP6	tumor necrosis factor, alpha-induced protein 6	Hs.437322
TNFRSF17	tumor necrosis factor receptor superfamily, member 17	Hs.2556
TNFSF10	tumor necrosis factor (ligand) superfamily, member 10	Hs.478275
TNFSF13B	tumor necrosis factor (ligand) superfamily, member 13b	Hs.525157
TOMM5	translocase of outer mitochondrial membrane 5 homolog (yeast)	Hs.130774
TPMT	thiopurine S-methyltransferase	Hs.444319
TRAF4	TNF receptor-associated factor 4	Hs.8375
TRAK2	trafficking protein, kinesin-binding 2	Hs.152774
TRAT1	T cell receptor-associated transmembrane adaptor 1	Hs.138701
TRBV27	T cell receptor beta variable 27	Hs.511522
TREM1	triggering receptor expressed on myeloid cells 1	Hs.283022
TRIM22	tripartite motif containing 22	Hs.501778
TRIM69	tripartite motif containing 69	Hs.489254
TRIQK	triple QxxK/R motif containing	Hs.440643
TSC22D3	TSC22 domain family, member 3	Hs.522074
TSPAN2	tetraspanin 2	Hs.310458
TSTA3	tissue specific transplantation antigen P35B	Hs.404119
TTC33	tetratricopeptide repeat domain 33	Hs.348915
TXN	thioredoxin	Hs.435136
TYMP	thymidine phosphorylase	Hs.592212
TYMS	thymidylate synthetase	Hs.592338
UBE2B	ubiquitin-conjugating enzyme E2B	Hs.612096
UBE2D1	ubiquitin-conjugating enzyme E2D 1	Hs.129683
UBE2E1	ubiquitin-conjugating enzyme E2E 1	Hs.164853
UBE2L6	ubiquitin-conjugating enzyme E2L 6	Hs.425777
UBTD2	ubiquitin domain containing 2	Hs.724634

UQCR11	ubiquinol-cytochrome c reductase, complex III subunit XI	Hs.8372
UQCRH /// UQCRHL	ubiquinol-cytochrome c reductase hinge protein /// ubiquinol-cytochrome c reductase hinge protein-like	Hs.481571
UQCRQ	ubiquinol-cytochrome c reductase, complex III subunit VII, 9.5 kDa	Hs.146602
USP15	ubiquitin specific peptidase 15	Hs.434951
UTS2	urotensin 2	Hs.715862
VAMP5	vesicle-associated membrane protein 5 (myobrevin)	Hs.534373
VBP1	von Hippel-Lindau-binding protein 1	Hs.436803
VRK2	vaccinia related kinase 2	Hs.631890
VSTM1	V-set and transmembrane domain containing 1	Hs.444431
WARS	tryptophanyl-tRNA synthetase	Hs.497599
WARS	tryptophanyl-tRNA synthetase	Hs.497599
WDFY1	WD repeat and FYVE domain containing 1	Hs.368359
WDFY3	WD repeat and FYVE domain containing 3	Hs.480116
WTAP	Wilms tumor 1-associated protein	Hs.446091
XAF1	XIAP-associated factor 1	Hs.441975
XK	X-linked Kx blood group (McLeod syndrome)	Hs.78919
YOD1	YOD1 OTU deubiquinating enzyme 1 homolog (*S. cerevisiae*)	Hs.567533
YPEL5	yippee-like 5 (*Drosophila*)	Hs.515890
ZBTB34	zinc finger and BTB domain containing 34	Hs.177633
ZBTB41	zinc finger and BTB domain containing 41	Hs.529439
ZC3H15	zinc finger CCCH-type containing 15	Hs.724407
ZCCHC10	zinc finger, CCHC domain containing 10	Hs.29700
ZDHHC18	zinc finger, DHHC-type containing 18	Hs.523710
ZNF117	zinc finger protein 117	Hs.250693
ZNF267	zinc finger protein 267	Hs.460645
ZNF467	zinc finger protein 467	Hs.112158
ZNF83	zinc finger protein 83	Hs.467210
ZNFX1-AS1	ZNFX1 antisense RNA 1 (non-protein coding)	Hs.356766
ZNHIT3	zinc finger, HIT-type containing 3	Hs.2210
ZWINT	ZW10 interactor	Hs.591363
