# Supplementary material for: Improvement in Human Immune Function with Changes in Intestinal Microbiota by Salacia reticulata Extract Ingestion: A Randomized Placebo-Controlled Trial
Source: PLoS One. 2015 Dec 2;10(12):e0142909. doi: 10.1371/journal.pone.0142909 (PMC4667990; doi:10.1371/journal.pone.0142909)
Supplement: S4 Table — (PDF) [file pone.0142909.s007.pdf]

Supplementary Data

Improvement in human immune function with changes in intestinal microbiota by *Salacia reticulata* extract ingestion

Yuriko Oda, Fumitaka Ueda, Masanori Utsuyama, Asuka Kamei, Chihaya Kakinuma, Keiko Abe, and Katsuiku Hirokawa

S4 Table. Down-regulated genes

The obtained data were normalized using the DFW method. Intergroup comparison of the DFW-normalized data was performed using the RP method. Probe sets with an FDR of <0.05 were extracted.

Gene Symbol	Gene Title	UniGene ID
A2M-AS1	A2M antisense RNA 1 (non-protein coding)	Hs.592432
AAK1	AP2-associated kinase 1	Hs.468878
ABCG1	ATP-binding cassette, sub-family G (WHITE), member 1	Hs.124649
ABHD2	abhydrolase domain containing 2	Hs.122337
ACSL1	acyl-CoA synthetase long-chain family member 1	Hs.406678
ACSL6 /// LOC100505572	acyl-CoA synthetase long-chain family member 6 /// uncharacterized LOC100505572	Hs.14945
ACTR2	ARP2 actin-related protein 2 homolog (yeast)	Hs.723952
ADAM28	ADAM metallopeptidase domain 28	Hs.174030
AFF3	AF4/FMR2 family, member 3	Hs.444414
AFF4	AF4/FMR2 family, member 4	Hs.519313
AKAP13	A kinase (PRKA) anchor protein 13	Hs.459211
AKAP2 /// PALM2-AKAP2	A kinase (PRKA) anchor protein 2 /// PALM2-AKAP2 readthrough	Hs.591908
ALDH5A1	aldehyde dehydrogenase 5 family, member A1	Hs.371723
ALMS1	Alstrom syndrome 1	Hs.184720
ANKRD11	ankyrin repeat domain 11	Hs.335003
ANKRD17	ankyrin repeat domain 17	Hs.724400
ANKRD36B	ankyrin repeat domain 36B	Hs.532921
ANXA11	annexin A11	Hs.530291
ARHGAP26	Rho GTPase-activating protein 26	Hs.654668
ARHGEF12	Rho guanine nucleotide exchange factor (GEF) 12	Hs.24598
ARL4C	ADP-ribosylation factor-like 4C	Hs.723194
ASCC2	activating signal cointegrator 1 complex subunit 2	Hs.517438
ASH1L	ash1 (absent, small, or homeotic)-like (*Drosophila*)	Hs.491060
ATF7IP	activating transcription factor 7-interacting protein	Hs.591151
ATG16L2	autophagy related 16-like 2 (*S. cerevisiae*)	Hs.653186
ATP2B4	ATPase, Ca++ transporting, plasma membrane 4	Hs.343522
ATP7A	ATPase, Cu++ transporting, alpha polypeptide	Hs.496414
ATP8B1	ATPase, aminophospholipid transporter, class I, type 8B, member 1	Hs.216623
ATXN1	ataxin 1	Hs.434961
ATXN7L3B	ataxin 7-like 3B	Hs.213541
AUTS2	autism susceptibility candidate 2	Hs.21631
BANK1	B-cell scaffold protein with ankyrin repeats 1	Hs.480400
BCL11A	B-cell CLL/lymphoma 11A (zinc finger protein)	Hs.370549
BCL11B	B-cell CLL/lymphoma 11B (zinc finger protein)	Hs.709690
BCL2L1	BCL2-like 1	Hs.516966
BCL2L11	BCL2-like 11 (apoptosis facilitator)	Hs.469658

BDP1	B double prime 1, subunit of RNA polymerase III transcription initiation factor IIIB	Hs.258272
BLVRB	biliverdin reductase B (flavin reductase (NADPH))	Hs.515785
BOD1L1	biorientation of chromosomes in cell division 1-like 1	Hs.444517
BPTF /// LOC146880	bromodomain PHD finger transcription factor /// Rho GTPase-activating protein 27 pseudogene	Hs.444200
BRD2	bromodomain containing 2	Hs.75243
BSG	basigin (Ok blood group)	Hs.501293
C10orf118	chromosome 10 open reading frame 118	Hs.159066
C12orf35	chromosome 12 open reading frame 35	Hs.445129
C1orf144	chromosome 1 open reading frame 144	Hs.252967
C22orf13	chromosome 22 open reading frame 13	Hs.9850
C5orf4	chromosome 5 open reading frame 4	Hs.713175
C9orf64	chromosome 9 open reading frame 64	Hs.208914
CARM1	coactivator-associated arginine methyltransferase 1	Hs.323213
CBL	Cbl proto-oncogene, E3 ubiquitin protein ligase	Hs.504096
CCL4	chemokine (C-C motif) ligand 4	Hs.75703
CCL5	chemokine (C-C motif) ligand 5	Hs.514821
CCND2	cyclin D2	Hs.376071
CCR3	chemokine (C-C motif) receptor 3	Hs.506190
CD180	CD180 molecule	Hs.87205
CD19	CD19 molecule	Hs.652262
CD2	CD2 molecule	Hs.523500
CD24	CD24 molecule	Hs.644105
CD247	CD247 molecule	Hs.156445
CD36	CD36 molecule (thrombospondin receptor)	Hs.120949
CD3G	CD3g molecule, gamma (CD3-TCR complex)	Hs.2259
CD47	CD47 molecule	Hs.446414
CD53	CD53 molecule	---
CD7	CD7 molecule	Hs.36972
CD79A	CD79a molecule, immunoglobulin-associated alpha	Hs.631567
CD79B	CD79b molecule, immunoglobulin-associated beta	Hs.89575
CD8A	CD8a molecule	Hs.85258
CD93	CD93 molecule	Hs.97199
CDC42	cell division cycle 42 (GTP-binding protein, 25 kDa)	Hs.467637
CELF2	CUGBP, Elav-like family member 2	Hs.309288
CEP350	centrosomal protein 350 kDa	Hs.413045
CFLAR	CASP8 and FADD-like apoptosis regulator	Hs.390736
CHD4	chromodomain helicase DNA-binding protein 4	Hs.162233
CHD7	chromodomain helicase DNA-binding protein 7	Hs.20395
CHL1-AS2	CHL1 antisense RNA 2 (non-protein coding)	Hs.570612
CHST12	carbohydrate (chondroitin 4) sulfotransferase 12	Hs.213088
CKAP2 /// IGLC1 /// IGLV1-44	Cytoskeleton-associated protein 2 /// Immunoglobulin lambda constant 1 (Mcg marker) /// Immunoglobulin lambda variable 1-44	---
CLIC3	chloride intracellular channel 3	Hs.64746

CLIP1	CAP-GLY domain containing linker protein 1	Hs.524809
CMC1	COX assembly mitochondrial protein 1 homolog (*S. cerevisiae*)	Hs.444724
CR1	complement component (3b/4b) receptor 1 (Knops blood group)	Hs.334019
CSDA	cold shock domain protein A	Hs.221889
CSF3R	colony stimulating factor 3 receptor (granulocyte)	Hs.524517
CST7	cystatin F (leukocystatin)	Hs.143212
CTSB	cathepsin B	Hs.520898
CTSW	cathepsin W	Hs.416848
CUX1	cut-like homeobox 1	Hs.654389
CX3CR1	chemokine (C-X3-C motif) receptor 1	Hs.78913
CXCR5	chemokine (C-X-C motif) receptor 5	Hs.113916
CXXC5	CXXC finger protein 5	Hs.189119
DAPP1	dual adaptor of phosphotyrosine and 3-phosphoinositides	Hs.436271
DCAF12	DDB1 and CUL4-associated factor 12	Hs.493750
DCUN1D1	DCN1, defective in cullin neddylation 1, domain containing 1 (*S. cerevisiae*)	Hs.104613
DDHD1	DDHD domain containing 1	Hs.513260
DDX17	DEAD (Asp-Glu-Ala-Asp) box helicase 17	Hs.528305
DDX24	DEAD (Asp-Glu-Ala-Asp) box polypeptide 24	Hs.510328
DDX6	DEAD (Asp-Glu-Ala-Asp) box helicase 6	Hs.408461
DDX60	DEAD (Asp-Glu-Ala-Asp) box polypeptide 60	Hs.591710
DHRS9	dehydrogenase/reductase (SDR family) member 9	Hs.179608
DHX9	DEAH (Asp-Glu-Ala-His) box polypeptide 9	Hs.191518
DOCK11	dedicator of cytokinesis 11	Hs.368203
DOCK8	dedicator of cytokinesis 8	Hs.132599
DTHD1	death domain containing 1	Hs.363407
DTX2P1-UPK3BP1-PMS2P11	DTX2P1-UPK3BP1-PMS2P11 readthrough (non-protein coding)	Hs.675888
DYNC1H1	dynein, cytoplasmic 1, heavy chain 1	Hs.649497
DYRK2	dual-specificity tyrosine-(Y)-phosphorylation regulated kinase 2	Hs.173135
DYSF	dysferlin, limb girdle muscular dystrophy 2B (autosomal recessive)	Hs.252180
E2F2	E2F transcription factor 2	Hs.194333
EBF1	Early B-cell factor 1	Hs.573143
EEPD1	endonuclease/exonuclease/phosphatase family domain containing 1	Hs.670591
EIF2AK1	eukaryotic translation initiation factor 2-alpha kinase 1	Hs.724455
ELF1	E74-like factor 1 (ets domain transcription factor)	Hs.135646
EMR3	egf-like module containing, mucin-like, hormone receptor-like 3	Hs.658712
EOMES	eomesodermin	Hs.591663
EP400	E1A-binding protein p400	Hs.595201
EPB41	erythrocyte membrane protein band 4.1 (elliptocytosis 1, RH-linked)	Hs.175437
EPHA4	EPH receptor A4	Hs.371218
ERAP2	endoplasmic reticulum aminopeptidase 2	Hs.482910
ETS1	v-ets erythroblastosis virus E26 oncogene homolog 1 (avian)	Hs.369438
EWSR1	Ewing sarcoma breakpoint region 1	Hs.374477
EZR	ezrin	Hs.487027
F2R	coagulation factor II (thrombin) receptor	Hs.482562

F5	coagulation factor V (proaccelerin, labile factor)	Hs.30054
FAIM3	Fas apoptotic inhibitory molecule 3	Hs.723317
FAM126B	family with sequence similarity 126, member B	Hs.24701
FAM129A	family with sequence similarity 129, member A	Hs.518662
FAM190B	family with sequence similarity 190, member B	Hs.461988
FAM210B	family with sequence similarity 210, member B	Hs.143736
FAM47A	family with sequence similarity 47, member A	Hs.143268
FAM65B	family with sequence similarity 65, member B	Hs.559459
FBXO9	F-box protein 9	Hs.216653
FCRL3	Fc receptor-like 3	Hs.292449
FCRL5	Fc receptor-like 5	Hs.415950
FCRLA	Fc receptor-like A	Hs.266331
FGFBP2	fibroblast growth factor-binding protein 2	Hs.98785
FLJ12120	uncharacterized LOC388439	Hs.515183
FNBP4	formin-binding protein 4	Hs.6834
FNIP1 /// RAPGEF6	folliculin-interacting protein 1 /// Rap guanine nucleotide exchange factor (GEF) 6	Hs.591273
FOXO1	forkhead box O1	Hs.370666
FOXP1	forkhead box P1	Hs.431498
FTX	FTX transcript, XIST regulator (non-protein coding)	Hs.349570
FUS	fused in sarcoma	Hs.46894
FUT11	fucosyltransferase 11 (alpha (1,3) fucosyltransferase)	---
FYB	FYN-binding protein	Hs.370503
GATAD1	GATA zinc finger domain containing 1	Hs.21145
GBP1	guanylate-binding protein 1, interferon-inducible	Hs.62661
GBP4	guanylate-binding protein 4	Hs.409925
GLG1	golgi glycoprotein 1	Hs.109731
GNLY	granulysin	Hs.105806
GON4L	gon-4-like (*C. elegans*)	Hs.656361
GPR56	G protein-coupled receptor 56	Hs.513633
GUSBP3 /// GUSBP9	glucuronidase, beta pseudogene 3 /// glucuronidase, beta pseudogene 9	---
GZMB	granzyme B (granzyme 2, cytotoxic T-lymphocyte-associated serine esterase 1)	Hs.1051
GZMM	granzyme M (lymphocyte met-ase 1)	Hs.465511
H2AFY	H2A histone family, member Y	---
HBM	hemoglobin, mu	Hs.647389
HBQ1	hemoglobin, theta 1	Hs.247921
HBZ	hemoglobin, zeta	Hs.585357
HDC	histidine decarboxylase	Hs.1481
HECTD1	HECT domain containing E3 ubiquitin protein ligase 1	Hs.708017
HIP1	Huntingtin-interacting protein 1	Hs.329266
HIPK2	Homeodomain-interacting protein kinase 2	Hs.724392
HIST1H1C	histone cluster 1, H1c	Hs.7644
HIST1H1E	histone cluster 1, H1e	Hs.248133
HLA-DPA1	major histocompatibility complex, class II, DP alpha 1	Hs.347270

HLA-DQA1 /// HLA-DQA2 /// LOC100507718 /// LOC100509457	major histocompatibility complex, class II, DQ alpha 1 /// major histocompatibility complex, class II, DQ alpha 2 /// HLA class II histocompatibility antigen, DQ alpha 1 chain-like /// HLA class II histocompatibility antigen, DQ alpha 1 chain-like	Hs.591798
HLA-DQB1 /// LOC100293977	major histocompatibility complex, class II, DQ beta 1 /// HLA class II histocompatibility antigen, DQ beta 1 chain-like	Hs.409934
HLA-DRB1 /// HLA-DRB3 /// HLA-DRB4 /// HLA-DRB5 /// LOC100507709 /// LOC100507714	major histocompatibility complex, class II, DR beta 1 /// major histocompatibility complex, class II, DR beta 3 /// major histocompatibility complex, class II, DR beta 4 /// major histocompatibility complex, class II, DR beta 5 /// HLA class II histocompatibility antigen, DRB1-7 beta chain-like /// HLA class II histocompatibility antigen, DRB1-7 beta chain-like	Hs.534322
HOPX	HOP homeobox	Hs.619396
HOXB1	homeobox B1	Hs.86937
HSP90AB1	heat shock protein 90 kDa alpha (cytosolic), class B member 1	Hs.509736
HSP90B1 /// MIR3652	heat shock protein 90 kDa beta (Grp94), member 1 /// microRNA 3652	Hs.192374
HSPA1A /// HSPA1B	heat shock 70 kDa protein 1A /// heat shock 70 kDa protein 1B	Hs.274402
HUWE1	HECT, UBA and WWE domain containing 1, E3 ubiquitin protein ligase	Hs.136905
ID2	inhibitor of DNA-binding 2, dominant negative helix-loop-helix protein	Hs.180919
IGF2BP2	insulin-like growth factor 2 mRNA-binding protein 2	Hs.35354
IGF2R	insulin-like growth factor 2 receptor	Hs.487062
IGHD	immunoglobulin heavy constant delta	---
IGHG1 /// IGHM	immunoglobulin heavy constant gamma 1 (G1m marker) /// immunoglobulin heavy constant mu	Hs.510635
IGHM	immunoglobulin heavy constant mu	---
IKZF3	IKAROS family zinc finger 3 (Aiolos)	Hs.444388
IL18RAP	interleukin 18 receptor accessory protein	Hs.158315
IL2RB	interleukin 2 receptor, beta	Hs.474787
IL32	interleukin 32	Hs.943
IL6ST	interleukin 6 signal transducer (gp130, oncostatin M receptor)	Hs.532082
IL7R	interleukin 7 receptor	Hs.591742
ILF3	interleukin enhancer-binding factor 3, 90 kDa	Hs.465885
INO80D	INO80 complex subunit D	Hs.445036
IQGAP1	IQ motif containing GTPase activating protein 1	Hs.430551
IRS2	insulin receptor substrate 2	Hs.442344
ITGA2B	integrin, alpha 2b (platelet glycoprotein IIb of IIb/IIIa complex, antigen CD41)	Hs.411312
ITGAL	integrin, alpha L (antigen CD11A (p180), lymphocyte function-associated antigen 1; alpha polypeptide)	Hs.174103
ITM2A	integral membrane protein 2A	Hs.17109
ITPKB	Inositol-trisphosphate 3-kinase B	Hs.659396
JAK1	Janus kinase 1	Hs.207538
JAZF1	JAZF zinc finger 1	Hs.368944
JMJD1C	jumonji domain containing 1C	Hs.413416
KAT6A	K(lysine) acetyltransferase 6A	Hs.491577

KCNA3	potassium voltage-gated channel, shaker-related subfamily, member 3	Hs.169948
KCTD12	potassium channel tetramerisation domain containing 12	Hs.644125
KDM4B	lysine (K)-specific demethylase 4B	Hs.654816
KDM4C	lysine (K)-specific demethylase 4C	Hs.709425
KDM5A	lysine (K)-specific demethylase 5A	Hs.76272
KDM6B	lysine (K)-specific demethylase 6B	Hs.223678
KIAA0430	KIAA0430	Hs.173524
KIR2DL1 /// KIR2DL2 /// KIR2DL3 /// KIR2DL4 /// KIR2DL5A /// KIR2DL5B /// KIR3DL3 /// KIR3DS1 /// LOC100287534 /// LOC100653050	killer cell immunoglobulin-like receptor, two domains, long cytoplasmic tail, 1 /// killer cell immunoglobulin-like receptor, two domains, long cytoplasmic tail, 2 /// killer cell immunoglobulin-like receptor, two domains, long cytoplasmic tail, 3 /// killer cell immunoglobulin-like receptor, two domains, long cytoplasmic tail, 4 /// killer cell immunoglobulin-like receptor, two domains, long cytoplasmic tail, 5A /// killer cell immunoglobulin-like receptor, two domains, long cytoplasmic tail, 5B /// killer cell immunoglobulin-like receptor three domains long cytoplasmic tail 3 /// killer cell immunoglobulin-like receptor, three domains, short cytoplasmic tail, 1 /// killer cell immunoglobulin-like receptor 2DL4-like /// killer cell immunoglobulin-like receptor 2DL2-like	Hs.720721
KIR2DL2	killer cell immunoglobulin-like receptor, two domains, long cytoplasmic tail, 2	Hs.659860
KIR2DL3	killer cell immunoglobulin-like receptor, two domains, long cytoplasmic tail, 3	Hs.654605
KIR2DS1 /// KIR2DS2 /// KIR2DS3 /// KIR2DS4 /// KIR2DS5 /// KIR3DL3	killer cell immunoglobulin-like receptor, two domains, short cytoplasmic tail, 1 /// killer cell immunoglobulin-like receptor, two domains, short cytoplasmic tail, 2 /// killer cell immunoglobulin-like receptor, two domains, short cytoplasmic tail, 3 /// killer cell immunoglobulin-like receptor, two domains, short cytoplasmic tail, 4 /// killer cell immunoglobulin-like receptor, two domains, short cytoplasmic tail, 5 /// killer cell immunoglobulin-like receptor three domains long cytoplasmic tail 3	Hs.512572
KIR2DS5	killer cell immunoglobulin-like receptor, two domains, short cytoplasmic tail, 5	Hs.714920
KIR3DL1	killer cell immunoglobulin-like receptor, three domains, long cytoplasmic tail, 1	Hs.645228
KIR3DL2 /// LOC727787	killer cell immunoglobulin-like receptor, three domains, long cytoplasmic tail, 2 /// killer cell immunoglobulin-like receptor, three domains, long cytoplasmic tail, 2-like	Hs.645228
KLF13	Kruppel-like factor 13	Hs.525752
KLHL6	kelch-like 6 (*Drosophila*)	Hs.659147
KLRC1 /// KLRC2	killer cell lectin-like receptor subfamily C, member 1 /// killer cell lectin-like receptor subfamily C, member 2	Hs.591157
KLRC3	killer cell lectin-like receptor subfamily C, member 3	Hs.654362
KLRC4	killer cell lectin-like receptor subfamily C, member 4	Hs.721094
KLRC4-KLRK1 /// KLRK1	KLRC4-KLRK1 readthrough /// killer cell lectin-like receptor subfamily K, member 1	Hs.387787
KLRD1	killer cell lectin-like receptor subfamily D, member 1	Hs.562457
KLRF1	killer cell lectin-like receptor subfamily F, member 1	Hs.183125
KLRG1	killer cell lectin-like receptor subfamily G, member 1	Hs.558446
KRAS	v-Ki-ras2 Kirsten rat sarcoma viral oncogene homolog	Hs.505033
LCP2	lymphocyte cytosolic protein 2 (SH2 domain containing leukocyte protein of 76 kDa)	Hs.304475
LINC00273 /// LINC00273	long intergenic non-protein coding RNA 273 /// long intergenic non-protein coding RNA 273	---
LINC00537	Long intergenic non-protein coding RNA 537	Hs.599821

LNPEP	leucyl/cystinyl aminopeptidase	Hs.527199
LOC100129518 /// SOD2	uncharacterized LOC100129518 /// superoxide dismutase 2, mitochondrial	Hs.487046
LOC100130175	uncharacterized LOC100130175	---
LOC100130872 /// SPON2	uncharacterized LOC100130872 /// spondin 2, extracellular matrix protein	Hs.635350
LOC100131564	uncharacterized LOC100131564	Hs.714927
LOC100132247 /// LOC100652992 /// LOC595101 /// LOC613037 /// LOC728888 /// NPIPL3	nuclear pore complex-interacting protein-like /// nuclear pore complex-interacting protein related gene /// uncharacterized LOC100652992 /// smg-1 homolog, phosphatidylinositol 3-kinase-related kinase (*C. elegans*) pseudogene /// nuclear pore complex-interacting protein pseudogene /// nuclear pore complex-interacting protein-like 3-like /// nuclear pore complex-interacting protein-like 3	Hs.611072
LOC100132247 /// LOC100652992 /// LOC613037 /// LOC728888 /// NPIPL3	nuclear pore complex-interacting protein-like /// nuclear pore complex-interacting protein related gene /// uncharacterized LOC100652992 /// nuclear pore complex-interacting protein pseudogene /// nuclear pore complex-interacting protein-like 3-like /// nuclear pore complex-interacting protein-like 3	Hs.720286
LOC100132832 /// PMS2L2 /// PMS2P5	PMS2 postmeiotic segregation increased 2 (*S. cerevisiae*) pseudogene /// postmeiotic segregation increased 2-like 2 pseudogene /// postmeiotic segregation increased 2 pseudogene 5	Hs.661055
LOC100134822	uncharacterized LOC100134822	Hs.721739
LOC100190986	uncharacterized LOC100190986	Hs.648439
LOC100272216	uncharacterized LOC100272216	Hs.662074
LOC100288142 /// NBPF1 /// NBPF10 /// NBPF8	neuroblastoma breakpoint family member /// neuroblastoma breakpoint family, member 1 /// neuroblastoma breakpoint family, member 10 /// neuroblastoma breakpoint family, member 8	Hs.533593
LOC100506032 /// NBPF10	neuroblastoma breakpoint family member 21-like /// neuroblastoma breakpoint family, member 10	Hs.515947
LOC100506060 /// LOC100506830 /// SMG1	SMG1 homolog, phosphatidylinositol 3-kinase-related kinase (C. elegans) pseudogene /// uncharacterized LOC100506830 /// smg-1 homolog, phosphatidylinositol 3-kinase-related kinase (C. elegans)	Hs.460179
LOC100506710	endogenous Bornavirus-like nucleoprotein 2 pseudogene	Hs.446271
LOC100506776	uncharacterized LOC100506776	Hs.173034
LOC100507645 /// MALAT1	uncharacterized LOC100507645 /// metastasis-associated lung adenocarcinoma transcript 1 (non-protein coding)	Hs.621695
LOC100507645 /// MALAT1	uncharacterized LOC100507645 /// metastasis-associated lung adenocarcinoma transcript 1 (non-protein coding)	Hs.621695
LOC100509323 /// NAIP	baculoviral IAP repeat containing protein 1-like /// NLR family, apoptosis inhibitory protein	Hs.654500
LOC100653017 /// MIR612 /// NEAT1	uncharacterized LOC100653017 /// microRNA 612 /// nuclear paraspeckle assembly transcript 1 (non-protein coding)	Hs.523789
LOC254896 /// TNFRSF10C	uncharacterized LOC254896 /// tumor necrosis factor receptor superfamily, member 10c, decoy without an intracellular domain	---

LOC254896 /// TNFRSF10C	uncharacterized LOC254896 /// tumor necrosis factor receptor superfamily, member 10c, decoy without an intracellular domain	Hs.655801
LOC283663	uncharacterized LOC283663	Hs.181297
LOC286087	uncharacterized LOC286087	Hs.202475
LOC339988	uncharacterized LOC339988	Hs.434746
LOC401463	uncharacterized LOC401463	Hs.651960
LOC441259 /// POLR2J2 /// POLR2J3 /// UPK3BL	PMS2 postmeiotic segregation increased 2 (*S. cerevisiae*)-like /// polymerase (RNA) II (DNA directed) polypeptide J2 /// polymerase (RNA) II (DNA directed) polypeptide J3 /// uroplakin 3B-like	Hs.610255
LOC642236	FSHD region gene 1 pseudogene	Hs.529357
LOC653562 /// SLC6A10P /// SLC6A8	sodium- and chloride-dependent creatine transporter 1-like /// solute carrier family 6 (neurotransmitter transporter, creatine), member 10, pseudogene /// solute carrier family 6 (neurotransmitter transporter, creatine), member 8	Hs.540696
LOC727820 /// LOC728855 /// LOC728875	uncharacterized LOC727820 /// uncharacterized LOC728855 /// uncharacterized LOC728875	Hs.724554
LPGAT1	lysophosphatidylglycerol acyltransferase 1	Hs.497674
LPP	LIM domain containing preferred translocation partner in lipoma	Hs.5724
LRRC37A /// LRRC37A2 /// LRRC37A3 /// LRRC37A4P	leucine rich repeat containing 37A /// leucine rich repeat containing 37, member A2 /// leucine rich repeat containing 37, member A3 /// leucine rich repeat containing 37, member A4, pseudogene	Hs.596756
LRRC37A4P	leucine rich repeat containing 37, member A4, pseudogene	Hs.596756
LUC7L3	LUC7-like 3 (*S. cerevisiae*)	Hs.130293
LYST	lysosomal trafficking regulator	Hs.532411
MAP4	microtubule-associated protein 4	Hs.517949
MAP4K4	mitogen-activated protein kinase kinase kinase kinase 4	Hs.431550
MBNL1	muscleblind-like splicing regulator 1	Hs.478000
MBNL3	muscleblind-like splicing regulator 3	Hs.105134
MBP	myelin basic protein	Hs.551713
MDM4	Mdm4 p53-binding protein homolog (mouse)	Hs.497492
MED13	mediator complex subunit 13	Hs.282678
MGAM	maltase-glucoamylase (alpha-glucosidase)	Hs.122785
MGC24103	uncharacterized MGC24103	Hs.664877
MIAT	myocardial infarction-associated transcript (non-protein coding)	Hs.517502
MIR181A2HG	MIR181A2 host gene (non-protein coding)	Hs.660412
MLL	myeloid/lymphoid or mixed-lineage leukemia (trithorax homolog, *Drosophila*)	Hs.258855
MLL3	myeloid/lymphoid or mixed-lineage leukemia 3	Hs.647120
MLL5	myeloid/lymphoid or mixed-lineage leukemia 5 (trithorax homolog, *Drosophila*)	Hs.592262
MLLT6	myeloid/lymphoid or mixed-lineage leukemia (trithorax homolog, *Drosophila*); translocated to, 6	Hs.91531
MME	membrane metallo-endopeptidase	Hs.307734
MMP15	matrix metallopeptidase 15 (membrane-inserted)	Hs.80343
MMP9	matrix metallopeptidase 9 (gelatinase B, 92 kDa gelatinase, 92 kDa type IV collagenase)	Hs.297413
MOB1A	MOB kinase activator 1A	Hs.724457

MRVI1	murine retrovirus integration site 1 homolog	Hs.501898
MYADM	myeloid-associated differentiation marker	Hs.380906
MYBL1	v-myb myeloblastosis viral oncogene homolog (avian)-like 1	Hs.445898
MYO6	myosin VI	Hs.149387
NAMPT	Nicotinamide phosphoribosyltransferase	Hs.489615
NCAM1	neural cell adhesion molecule 1	Hs.503878
NCKAP1L	NCK-associated protein 1-like	Hs.182014
NCOA2	nuclear receptor coactivator 2	Hs.446678
NFAT5	nuclear factor of activated T-cells 5, tonicity-responsive	Hs.371987
NFATC2	nuclear factor of activated T-cells, cytoplasmic, calcineurin-dependent 2	Hs.713650
NFATC3	nuclear factor of activated T-cells, cytoplasmic, calcineurin-dependent 3	Hs.632209
NFIX	nuclear factor I/X (CCAAT-binding transcription factor)	Hs.257970
NINJ2	ninjurin 2	Hs.656450
NIPBL	Nipped-B homolog (*Drosophila*)	Hs.481927
NKG7	natural killer cell group 7 sequence	Hs.10306
NKTR	natural killer-tumor recognition sequence	Hs.529509
NOV	nephroblastoma overexpressed	Hs.235935
NPAT	nuclear protein, ataxia-telangiectasia locus	Hs.171061
NPIPL2	nuclear pore complex-interacting protein-like 2	Hs.448833
NPL	N-acetylneuraminate pyruvate lyase (dihydrodipicolinate synthase)	Hs.496969
NPRL3	nitrogen permease regulator-like 3 (*S. cerevisiae*)	Hs.19699
NPW	neuropeptide W	Hs.233533
NUCKS1	nuclear casein kinase and cyclin-dependent kinase substrate 1	Hs.213061
NUMB	numb homolog (*Drosophila*)	Hs.654609
NXPE3	neurexophilin and PC-esterase domain family, member 3	Hs.595933
ODC1	ornithine decarboxylase 1	Hs.467701
OLIG1	oligodendrocyte transcription factor 1	Hs.56663
ORMDL1	ORM1-like 1 (*S. cerevisiae*)	Hs.700632
OSBP2	oxysterol-binding protein 2	Hs.517546
OSBPL10	oxysterol-binding protein-like 10	Hs.150122
OSBPL8	oxysterol-binding protein-like 8	Hs.430849
P2RX5	purinergic receptor P2X, ligand-gated ion channel, 5	Hs.408615
PADI4	peptidyl arginine deiminase, type IV	Hs.522969
PAPD4	PAP-associated domain containing 4	---
PAX5	paired box 5	Hs.654464
PCDH9	protocadherin 9	Hs.654709
PCNP	PEST proteolytic signal containing nuclear protein	Hs.275865
PDIA3	protein disulfide isomerase family A, member 3	Hs.591095
PDPR	pyruvate dehydrogenase phosphatase regulatory subunit	Hs.724494
PDZK1IP1	PDZK1-interacting protein 1	Hs.431099
PECAM1	platelet/endothelial cell adhesion molecule 1	Hs.376675
PGF	placental growth factor	Hs.252820
PHF3	PHD finger protein 3	Hs.348921
PIK3CG	phosphoinositide-3-kinase, catalytic, gamma polypeptide	Hs.32942
PIP4K2A	phosphatidylinositol-5-phosphate 4-kinase, type II, alpha	Hs.57079

PLEKHA1	pleckstrin homology domain containing, family A (phosphoinositide binding specific) member 1	Hs.643512
PLEKHA2	pleckstrin homology domain containing, family A (phosphoinositide binding specific) member 2	Hs.369123
PLEKHG3	pleckstrin homology domain containing, family G (with RhoGef domain) member 3	Hs.509637
PLGLA /// PLGLB1 /// PLGLB2	plasminogen-like A /// plasminogen-like B1 /// plasminogen-like B2	Hs.652174
POLR2J2 /// POLR2J3 /// UPK3BL	polymerase (RNA) II (DNA directed) polypeptide J2 /// polymerase (RNA) II (DNA directed) polypeptide J3 /// uroplakin 3B-like	Hs.610255
POLR2J4	polymerase (RNA) II (DNA directed) polypeptide J4, pseudogene	Hs.657028
POU2AF1	POU class 2-associating factor 1	Hs.654525
POU2F2	POU class 2 homeobox 2	Hs.649101
PPDPF	pancreatic progenitor cell differentiation and proliferation factor homolog (zebrafish)	Hs.79625
PPM1K	protein phosphatase, Mg2+/Mn2+ dependent, 1K	Hs.291000
PPP1R12B	protein phosphatase 1, regulatory subunit 12B	Hs.444403
PPP1R16B	protein phosphatase 1, regulatory subunit 16B	Hs.45719
PRDM2	PR domain containing 2, with ZNF domain	Hs.371823
PRDX2	peroxiredoxin 2	Hs.432121
PRDX6	peroxiredoxin 6	Hs.120
PRF1	perforin 1 (pore forming protein)	Hs.2200
PRKCB	protein kinase C, beta	Hs.460355
PRKCH	protein kinase C, eta	Hs.333907
PRMT2	protein arginine methyltransferase 2	Hs.661229
PRPF6	PRP6 pre-mRNA processing factor 6 homolog (*S. cerevisiae*)	Hs.31334
PRR5	proline rich 5 (renal)	Hs.102336
PRRC2C	proline-rich coiled-coil 2C	Hs.494614
PRSS23	protease, serine, 23	Hs.25338
PSME4	proteasome (prosome, macropain) activator subunit 4	Hs.413801
PSMF1	proteasome (prosome, macropain) inhibitor subunit 1 (PI31)	Hs.471917
PTCH1	patched 1	Hs.494538
PTEN	phosphatase and tensin homolog	Hs.500466
PTGDR	prostaglandin D2 receptor (DP)	Hs.306831
PTGDS	prostaglandin D2 synthase 21 kDa (brain)	Hs.446429
PTPN4	protein tyrosine phosphatase, non-receptor type 4 (megakaryocyte)	Hs.469809
PTPRC	protein tyrosine phosphatase, receptor type, C	Hs.654514
PTPRO	protein tyrosine phosphatase, receptor type, O	Hs.160871
PXK	PX domain containing serine/threonine kinase	Hs.190544
PYHIN1	pyrin and HIN domain family, member 1	Hs.710248
RAB11FIP1	RAB11 family-interacting protein 1 (class I)	Hs.191179
RAB18	RAB18, member RAS oncogene family	Hs.406799
RABGAP1L	RAB GTPase activating protein 1-like	Hs.585378
RAD23A	RAD23 homolog A (*S. cerevisiae*)	Hs.643267
RANBP2	RAN-binding protein 2	Hs.199561

RAPGEF2	Rap guanine nucleotide exchange factor (GEF) 2	Hs.723853
RASEF	RAS and EF-hand domain containing	Hs.657750
RASGRP1	RAS guanyl releasing protein 1 (calcium and DAG-regulated)	Hs.591127
RBL2	retinoblastoma-like 2 (p130)	Hs.513609
RBM26	RNA-binding motif protein 26	Hs.558528
RBM39	RNA-binding motif protein 39	---
RBM5	RNA-binding motif protein 5	Hs.439480
RHOH	ras homolog family member H	Hs.654594
RIOK3	RIO kinase 3 (yeast)	Hs.445511
RN45S	45S pre-ribosomal RNA	Hs.370699
RNASET2	ribonuclease T2	Hs.529989
RNF213	ring finger protein 213	Hs.195642
RORA	RAR-related orphan receptor A	Hs.560343
RUNX3	runt-related transcription factor 3	Hs.170019
S1PR5	sphingosine-1-phosphate receptor 5	Hs.501561
SAMD9L	sterile alpha motif domain containing 9-like	Hs.489118
SART3	squamous cell carcinoma antigen recognized by T cells 3	Hs.584842
SEC14L1	SEC14-like 1 (*S. cerevisiae*)	Hs.464184
SECISBP2L	SECIS-binding protein 2-like	Hs.9997
SETD2	SET domain containing 2	Hs.517941
SETX	senataxin	Hs.460317
SF1	splicing factor 1	Hs.502829
SF3B1	splicing factor 3b, subunit 1, 155 kDa	Hs.632554
SH2D1A	SH2 domain containing 1A	Hs.349094
SH2D1B	SH2 domain containing 1B	Hs.350581
SH2D2A	SH2 domain containing 2A	Hs.103527
SIGLEC10	sialic acid-binding Ig-like lectin 10	Hs.249741
SKI	v-ski sarcoma viral oncogene homolog (avian)	Hs.656507
SLAIN2	SLAIN motif family, member 2	Hs.479677
SLAMF7	SLAM family member 7	Hs.517265
SLC11A1	solute carrier family 11 (proton-coupled divalent metal ion transporters), member 1	Hs.591607
SLC16A6	solute carrier family 16, member 6 (monocarboxylic acid transporter 7)	Hs.42645
SLC24A4	solute carrier family 24 (sodium/potassium/calcium exchanger), member 4	Hs.510281
SLC38A1	solute carrier family 38, member 1	Hs.533770
SLC6A8	solute carrier family 6 (neurotransmitter transporter, creatine), member 8	Hs.540696
SLC8A1	solute carrier family 8 (sodium/calcium exchanger), member 1	Hs.31961
SLFN5	schlafen family member 5	Hs.709347
SMARCA2	SWI/SNF related, matrix-associated, actin-dependent regulator of chromatin, subfamily a, member 2	Hs.298990
SMCHD1	structural maintenance of chromosomes flexible hinge domain containing 1	Hs.8118
SMOX	spermine oxidase	Hs.433337
SNX8	Sorting nexin 8	Hs.594383
SON	SON DNA-binding protein	Hs.517262
SORL1	sortilin-related receptor, L(DLR class) A repeats containing	Hs.368592
SOS1	son of sevenless homolog 1 (*Drosophila*)	Hs.709893

SPDYE2	Speedy homolog E2 (*Xenopus laevis*)	Hs.656428
SPEN	spen homolog, transcriptional regulator (*Drosophila*)	Hs.724378
SPIB	Spi-B transcription factor (Spi-1/PU.1 related)	Hs.437905
SPN	sialophorin	Hs.632188
SPOCK2	sparc/osteonectin, cwcv and kazal-like domains proteoglycan (testican) 2	Hs.523009
SRRM1	serine/arginine repetitive matrix 1	Hs.18192
SRRM2	serine/arginine repetitive matrix 2	Hs.433343
SRSF7	serine/arginine-rich splicing factor 7	Hs.309090
SSR3	signal sequence receptor, gamma (translocon-associated protein gamma)	Hs.518346
STAT4	signal transducer and activator of transcription 4	Hs.80642
STK4	serine/threonine kinase 4	Hs.472838
STRADB	STE20-related kinase adaptor beta	Hs.652338
STRBP	spermatid perinuclear RNA-binding protein	Hs.694157
STYX	serine/threonine/tyrosine-interacting protein	Hs.364980
SULF2	sulfatase 2	Hs.162016
SUN2	Sad1 and UNC84 domain containing 2	Hs.517622
SWAP70	SWAP switching B-cell complex 70 kDa subunit	Hs.153026
SYNE1	spectrin repeat containing, nuclear envelope 1	Hs.12967
SYNE2	spectrin repeat containing, nuclear envelope 2	Hs.525392
SYTL2	synaptotagmin-like 2	Hs.369520
SYTL3	synaptotagmin-like 3	Hs.436977
TARP	TCR gamma alternate reading frame protein	Hs.534032
TBL1X	transducin (beta)-like 1X-linked	Hs.495656
TBX21	T-box 21	Hs.272409
TBX3	T-box 3	Hs.129895
TCF25	transcription factor 25 (basic helix-loop-helix)	Hs.415342
TCF4	transcription factor 4	Hs.605153
TCL1A	T-cell leukemia/lymphoma 1A	Hs.2484
TESC	tescalcin	Hs.525709
TET2	tet methylcytosine dioxygenase 2	Hs.367639
TGFBR3	transforming growth factor, beta receptor III	Hs.482390
TGOLN2	trans-golgi network protein 2	Hs.593382
THRAP3	thyroid hormone receptor-associated protein 3	Hs.160211
TLE3	transducin-like enhancer of split 3 (E(sp1) homolog, *Drosophila*)	Hs.287362
TMCC1	transmembrane and coiled-coil domain family 1	---
TMX4	thioredoxin-related transmembrane protein 4	Hs.169358
TNFRSF10C	tumor necrosis factor receptor superfamily, member 10c, decoy without an intracellular domain	Hs.655801
TNRC6B	trinucleotide repeat containing 6B	Hs.372082
TNS1	tensin 1	Hs.471381
TOB2	transducer of ERBB2, 2	Hs.723848
TOX	thymocyte selection-associated high mobility group box	Hs.491805
TP53INP2	tumor protein p53 inducible nuclear protein 2	Hs.516994
TPR	translocated promoter region, nuclear basket protein	Hs.279640
TRAF3IP3	TRAF3-interacting protein 3	Hs.147434
TRAK2	trafficking protein, kinesin-binding 2	Hs.152774

TRDV3	T cell receptor delta variable 3	---
TRIM38	tripartite motif containing 38	Hs.715869
TRIM58	tripartite motif containing 58	Hs.323858
TSPAN5	tetraspanin 5	Hs.118118
TTC3 /// TTC3P1	tetratricopeptide repeat domain 3 /// tetratricopeptide repeat domain 3 pseudogene 1	Hs.368214
TUBB2A /// TUBB2B	tubulin, beta 2A class IIa /// tubulin, beta 2B class IIb	Hs.724372
TXK	TXK tyrosine kinase	Hs.479669
UBE2B	ubiquitin-conjugating enzyme E2B	Hs.612096
UBE2H	ubiquitin-conjugating enzyme E2H	Hs.643548
UBE2O	ubiquitin-conjugating enzyme E2O	Hs.16130
UBLCP1	ubiquitin-like domain containing CTD phosphatase 1	Hs.591733
UBN1	ubinuclein 1	Hs.440219
UBN2	ubinuclein 2	Hs.153458
UBXN6	UBX domain protein 6	Hs.435255
UCP2	uncoupling protein 2 (mitochondrial, proton carrier)	Hs.80658
UHMK1	U2AF homology motif (UHM) kinase 1	Hs.127310
USP34	ubiquitin specific peptidase 34	Hs.644708
VCAN	versican	Hs.643801
VPS13B	vacuolar protein sorting 13 homolog B (yeast)	Hs.191540
VTI1A	vesicle transport through interaction with t-SNAREs homolog 1A (yeast)	Hs.194554
WASF2	WAS protein family, member 2	Hs.590909
WHSC1L1	Wolf-Hirschhorn syndrome candidate 1-like 1	Hs.608111
WNK1	WNK lysine deficient protein kinase 1	Hs.719460
WSB1	WD repeat and SOCS box containing 1	Hs.446017
YME1L1	YME1-like 1 (*S. cerevisiae*)	Hs.74647
ZAP70	zeta-chain (TCR)-associated protein kinase 70 kDa	Hs.234569
ZBTB38	zinc finger and BTB domain containing 38	Hs.518301
ZBTB40	zinc finger and BTB domain containing 40	Hs.418966
ZC3H7B	zinc finger CCCH-type containing 7B	Hs.592188
ZFAND6	Zinc finger, AN1-type domain 6	Hs.596679
ZFP36L1	zinc finger protein 36, C3H type-like 1	Hs.85155
ZFR	zinc finger RNA-binding protein	Hs.435231
ZFYVE16	zinc finger, FYVE domain containing 16	---
ZMYM2	zinc finger, MYM-type 2	Hs.644041
ZNF160	zinc finger protein 160	Hs.655967
ZNF207	zinc finger protein 207	Hs.500775
ZNF345	zinc finger protein 345	Hs.362324
ZNF493	zinc finger protein 493	Hs.656558
ZNF609	zinc finger protein 609	Hs.595451
ZNF641	zinc finger protein 641	Hs.23492
ZNF800	zinc finger protein 800	Hs.159006
